# Supplementary material for: Wavefunction matching for solving quantum many-body problems
Source: Nature. 2024 May 15;630(8015):59–63. doi: 10.1038/s41586-024-07422-z (PMC11153134; doi:10.1038/s41586-024-07422-z)
Supplement: Supplementary file 1 — Supplementary Sections 1–18, including Supplementary Figs. 1–16, Tables 1–19 and References. [file 41586_2024_7422_MOESM1_ESM.pdf]

nature portfolio

https://doi.org/10.1038/s41586-024-07422-z

Supplementary information

Wavefunction matching for solving quantum many-body problems

In the format provided by the
authors and unedited

Supplementary Information

The material in Supplementary Information is organized as follows. The first two sections are about nuclear lattice effective field theory and the lattice operators used. The next eight sections focus on wavefunction matching. We discuss theoretical concepts, benchmark tests, and the extension to continuous space. Then follows a section on uncertainties due to the chiral interactions. After this, we have four sections on aspects of chiral effective field theory, three-nucleon interactions, and new chiral interactions using wavefunction matching. We then conclude with a section on charge radii, a section presenting tables with details of the lattice results, and a section on three-nucleon interaction contributions to the binding energies. All quoted error bars are by default one-sigma error bars.

S1 Nuclear Lattice Effective Field Theory

Nuclear lattice effective field theory (NLEFT)[1,2] combines the frameworks of chiral effective field theory (χEFT) for the forces between nucleons, lattice field theory, and stochastic Monte Carlo algorithms. Each of these forms a cornerstone of the modern approach to strongly interacting many-fermion systems in many fields of study, notably nuclear, particle, condensed matter and atomic physics. By a unique merger of these cornerstones, NLEFT has matured into a leading framework for the investigation of nuclear structure properties.

Building upon the early developments,[3] NLEFT has extended its reach to light and medium-mass nuclei, mostly of even-even type. This has enabled detailed predictions[4–6] of nuclear structure properties, in particular the binding energy for ^{3}H and ^{4}He at NNLO,[7] as well as binding energies for isotopic chains with $Z = 1,2,4,6$ and 8,[8] together with root-mean-square radii and proton and neutron densities. Moreover, NLEFT has given further impulse to the investigation of α-clustering in nuclear matter. The analyses of the Hoyle state[9,10] and further low-lying excited states[10,11] of ^{12}C, as well as the study of the structure and EM properties of the 0_1^+, the 0_2^+ and 2_1^+ states of ^{16}O,[12] and the prediction of ground-state properties of even-even self-conjugate nuclei with $Z \leq 14$,[13] are noteworthy in this respect.

An immediate strength of NLEFT is the favorable computational scaling with nucleon number A, as the A-body Hamiltonian in NLEFT is diagonalized stochastically by means of an auxiliary field Monte Carlo (AFMC) algorithm. The two-body, three-body, and pion exchange interactions are described by auxiliary fields, which are sampled at each step in the AFMC algorithm. Energy eigenvalues are obtained using the adiabatic projection method, whereby transition amplitudes mediated by the Schrödinger time evolution operator between trial (Slater-determinant) states are constructed. In practical AFMC calculations of NLEFT, the temporal evolution of the trial states is discretized into L_t equal steps, separated by a temporal lattice spacing a_t. Hence, the time-evolution operator is subdivided into a sequence of L_t transfer matrices (Trotter decomposition).

On the one hand, NLEFT is firmly grounded in low-energy QCD, as the interactions between nucleons are described by the NNLO or N3LO Lagrangians of χEFT. The resulting Hamiltonian is formulated with a finite cubic lattice regulator, and avoids the need for *pre-diagonalization* techniques such as the similarity renormalization group (SRG).[14] On the other hand, it is known that the AFMC implementation of the full NNLO or N3LO χEFT Hamiltonian produces a severe sign problem, which eventually compromises the effectiveness of the numerical technique.

A firmly-rooted remedy in the NLEFT literature[1] entails the replacement of the χEFT transfer matrices in the initial and the final L_{t_o} time steps by pionless $SU(4)$-symmetric transfer matrices. It follows that the full χEFT Hamiltonian acts only in the middle $L_{t_i} \equiv L_t - L_{t_o}$ time steps through the transfer matrices, whereas Wigner's SU(4) one acts as a low-energy filter[1] at the boundaries of the time interval. This procedure extends to the expectation value of any operator representing a physical observable, inserted in the midpoint of the chain of transfer-matrices. The positiveness of the transfer matrices, where Wigner's SU(4) action is used, is the major advantage of the method, and is guaranteed for systems with even number of nucleons and either spin-singlet or isospin-singlet quantum numbers.[1] However, distortions in the energy eigenvalues as a result of the usage of an isospin-preserving SU(4) Lagrangian in the action do appear in the form of lower bounds.[1] Although remedies such as eigenvector continuation[15] or symmetry-sign extrapolation[16] would allow for a broader use of the χEFT transfer matrix, in this work we tackle the problem by using the method of wavefunction matching.

As in previous studies using NLEFT, we use a low-energy scheme to simplify the operators obtained in chiral effective field theory. The two-pion exchange interactions have an asymptotic spatial dependence proportional to $e^{-2M_\pi r}$ times power law factors of $1/r$. For our lattice spacing of $a = 1.32$ fm, the details of the two-pion exchange potential are not fully resolved. We therefore treat two-pion exchange interactions as well as higher-pion exchange interactions in the same manner as the short-range contact interactions. Within this low-energy scheme of chiral effective field theory, our lattice calculations include all two-nucleon and three-nucleon interactions up to $O(Q^4)$ or next-to-next-to-next-to-leading order (N3LO). The additional three-nucleon interactions used in our calculations correspond with a particular choice of the local regulators used for the three-nucleon interactions. We have not included four-nucleon interactions which also first appear at N3LO. In short, we are implementing chiral effective field theory at N3LO in the low-energy scheme, with special local regulators for the three-nucleon interactions and without four-nucleon interactions.

In this paper, we perform lattice Monte Carlo simulations of light nuclei, medium-mass nuclei up to $A = 58$, neutron matter up to $A = 80$, and nuclear matter up to $A = 160$. The method we employ can be used for calculations of heavier nuclei with $A > 58$ but may also benefit from increasing the efficiency of the computational algorithms and revisiting the parameterization of the three-body interactions.

S2 Lattice Operators

S2.1 Simple Hamiltonian

We present the details of our simple Hamiltonian H^S. We construct the Hamiltonian using a χEFT interaction at leading order,

$$H^S = K + \frac{c_{\mathrm{SU}(4)}}{2} \sum_{\vec{n}} : \left[\tilde{\rho}^{(1)}(\vec{n})\right]^2 : + \frac{c_I}{2} \sum_{I,\vec{n}} : \left[\tilde{\rho}_I^{(1)}(\vec{n})\right]^2 : + V_{\mathrm{OPE}}^{\Lambda_\pi}, \tag{S1}$$

where K is the kinetic energy term with nucleon mass $m = 938.92$ MeV, the :: symbol indicates normal ordering, and $\tilde{\rho}^{(d)}$ and $\tilde{\rho}_I^{(d)}$ are density operators that are smeared both locally and non-locally,

$$\tilde{\rho}^{(d)}(\vec{n}) = \sum_{i,j=0,1} \tilde{a}^{\dagger}_{i,j}(\vec{n})\, \tilde{a}_{i,j}(\vec{n}) + s_{\mathrm{L}} \sum_{|\vec{n}-\vec{n}'|^2=1}^{d} \sum_{i,j=0,1} \tilde{a}^{\dagger}_{i,j}(\vec{n}')\, \tilde{a}_{i,j}(\vec{n}'), \tag{S2}$$

$$\tilde{\rho}_I^{(d)}(\vec{n}) = \sum_{i,j,j'=0,1} \tilde{a}^{\dagger}_{i,j}(\vec{n}) \,[\tau_I]_{j,j'}\, \tilde{a}_{i,j'}(\vec{n}) + s_{\mathrm{L}} \sum_{|\vec{n}-\vec{n}'|^2=1}^{d} \sum_{i,j,j'=0,1} \tilde{a}^{\dagger}_{i,j}(\vec{n}') \,[\tau_I]_{j,j'}\, \tilde{a}_{i,j'}(\vec{n}'). \tag{S3}$$

For the kinetic energy operator we use fast Fourier transforms to produce the exact $p^2/(2m)$ dependence on the particle momenta. The smeared annihilation and creation operators, $\tilde{a}$ and $\tilde{a}^{\dagger}$, have with spin $i = 0,1$ (up, down) and isospin $j = 0,1$ (proton, neutron) indices,

$$\tilde{a}_{i,j}(\vec{n}) = a_{i,j}(\vec{n}) + s_{\mathrm{NL}} \sum_{|\vec{n}'-\vec{n}|=1} a_{i,j}(\vec{n}'). \tag{S4}$$

Throughout our calculations we use local smearing parameter $s_{\mathrm{L}} = 0.07$ and nonlocal smearing parameter $s_{\mathrm{NL}} = 0.5$. These parameters are similar to the values used in Ref.[17] and Ref.[18] for the same lattice spacing. Both s_L and s_{NL} contribute to the range of the two-nucleon interaction. However, s_L has a special role because the local smearing has a large impact on the $\alpha\alpha$ interaction, which in turn is important for nuclear binding. The values of these parameters are essential for dictating the many-body properties of the simple Hamiltonian H^S. The success of wavefunction matching for the many-body system relies on H^S having the correct basic features of the many-body system of interest, albeit with lower fidelity.

In addition to the short-range SU(4) symmetric interaction, we also have a long-range one-pion-exchange (OPE) potential at leading order χEFT interaction. We define our one-pion-exchange potential following a recently developed regularization method,[19]

$$V_{\mathrm{OPE}}^{\Lambda_\pi} = -\frac{g_A^2}{8 f_\pi^2} \sum_{\mathbf{n}',\mathbf{n},S',S,I} : \rho^{(0)}_{S',I}(\vec{n}') f_{S',S}(\vec{n}'-\vec{n}) \rho^{(0)}_{S,I}(\vec{n}) :, \tag{S5}$$

$$V_{\mathrm{C}\pi}^{\Lambda_\pi} = -C_\pi \frac{g_A^2}{8 f_\pi^2} \sum_{\mathbf{n}',\mathbf{n},S,I} : \rho^{(0)}_{S,I}(\vec{n}') f^{\pi}(\vec{n}'-\vec{n}) \rho^{(0)}_{S,I}(\vec{n}) : . \tag{S6}$$

Here f^π is a local regulator in momentum space defined as

$$f^{\pi}(\vec{n}'-\vec{n}) = \frac{1}{L^3} \sum_{\vec{q}} e^{-i\vec{q}\cdot(\vec{n}'-\vec{n}) - (\vec{q}^2+M_\pi^2)/\Lambda_\pi^2}, \tag{S7}$$

$f_{S',S}$ is the locally-regulated pion correlation function,

$$f_{S',S}(\vec{n}'-\vec{n}) = \frac{1}{L^3} \sum_{\vec{q}} \frac{q_{S'} q_S \, e^{-i\vec{q}\cdot(\vec{n}'-\vec{n}) - (\vec{q}^2+M_\pi^2)/\Lambda_\pi^2}}{\vec{q}^2 + M_\pi^2}, \tag{S8}$$

and

$$C_\pi = -\frac{\Lambda_\pi(\Lambda_\pi^2 - 2M_\pi^2) + 2\sqrt{\pi}M_\pi^3 \exp(M_\pi^2/\Lambda_\pi^2)\mathrm{erfc}(M_\pi/\Lambda_\pi)}{3\Lambda_\pi^3}\,, \tag{S9}$$

with $g_A = 1.287$ the axial-vector coupling constant (adjusted to account for the Goldberger-Treiman discrepancy),[20] $f_\pi = 92.2$ MeV the pion decay constant and $M_\pi = 134.98$ MeV the pion mass. The term given in Eq. (S6) is a counterterm introduced to remove the short-distance admixture in the one-pion-exchange potential.[19] In our simple Hamiltonian, we set $\Lambda_\pi = 180$ MeV and $C_\pi = 0$, and we compute the difference $V_{\rm OPE}^{\Lambda_\pi=300} - V_{\rm OPE}^{\Lambda_\pi=180}$ and the OPEP counterterm $V_{C_\pi}^{\Lambda_\pi}$ perturbatively. Here we use the notation

$$\rho^{(d)}(\vec{n}) = \sum_{i,j=0,1} a^\dagger_{i,j}(\vec{n})\,a_{i,j}(\vec{n}) + s_{\rm L} \sum_{|\vec{n}-\vec{n}'|^2=1}^{d} \sum_{i,j=0,1} a^\dagger_{i,j}(\vec{n}')\,a_{i,j}(\vec{n}'). \tag{S10}$$

and

$$\rho^{(d)}_{S,I}(\vec{n}) = \sum_{i,j,i',j'=0,1} a^\dagger_{i,j}(\vec{n})\,[\sigma_S]_{ii'}\,[\sigma_I]_{jj'}\,a_{i',j'}(\vec{n}) + s_{\rm L} \sum_{|\vec{n}-\vec{n}'|^2=1}^{d} \sum_{i,j,i',j'=0,1} a^\dagger_{i,j}(\vec{n}')\,[\sigma_S]_{ii'}\,[\sigma_I]_{jj'}\,a_{i',j'}(\vec{n}') \tag{S11}$$

for the density operators.

S2.2 Hamiltonian at N3LO (next-to-next-to-next-to-leading order)

We now give the details of our realistic Hamiltonian H. Let us define the functions

$$f_S^{\pi}(\vec{n}'-\vec{n}) = \frac{1}{L^3}\sum_{\vec{q}} e^{-i\vec{q}\cdot(\vec{n}'-\vec{n})-(\vec{q}^2+M_\pi^2)/\Lambda_\pi^2}\,q_S \tag{S12}$$

and

$$f_S^{\pi\pi}(\vec{n}'-\vec{n}) = \frac{1}{L^3}\sum_{\vec{q}} \frac{e^{-i\vec{q}\cdot(\vec{n}'-\vec{n})-(\vec{q}^2+M_\pi^2)/\Lambda_\pi^2}}{\vec{q}^2+M_\pi^2}\,q_S\,. \tag{S13}$$

We define the Hamiltonian H using χEFT interactions at N3LO,

$$H = K + V_{\rm OPE}^{\Lambda_\pi} + V_{C_\pi}^{\Lambda_\pi} + V_{\rm Coulomb} + V_{\rm 3N}^{\rm Q^3} + V_{\rm 2N}^{\rm Q^4} + W_{\rm 2N}^{\rm Q^4} + V_{\rm 2N,WFM}^{\rm Q^4} + W_{\rm 2N,WFM}^{\rm Q^4}. \tag{S14}$$

$V_{\rm OPE}^{\Lambda_\pi}$ and $V_{C_\pi}^{\Lambda_\pi}$ are defined in Eqs. (S5) and (S6) with $\Lambda_\pi = 300$ MeV. $V_{\rm Coulomb}$ is the Coulomb interaction, $V_{\rm 3N}^{\rm Q^3}$ is the 3N potential, $V_{\rm 2N}^{\rm Q^4}$ is the 2N short-range interaction at N3LO of χEFT, $W_{\rm 2N}^{\rm Q^4}$ is the 2N Galilean invariance restoration (GIR) interaction at N3LO of χEFT, $V_{\rm 2N,WFM}^{\rm Q^4}$ is the wavefunction matching interaction defined as $H'-H$, and $W_{\rm 2N,WFM}^{\rm Q^4}$ is the GIR correction of the wavefunction matching interaction.

For the details of the Coulomb interaction and the two-nucleon (2N) short-range interactions we refer the reader to Ref.[21] The three-nucleon (3N) interactions at $\rm Q^3$ consists of a contact potential, one-pion exchange potential, and two-pion exchange potential,[22–24] and in this work we defined two additional SU(4) symmetric potentials denoted by $V_{c_E}^{(l)}$ and $V_{c_E}^{(t)}$. Therefore, the three-nucleon interactions at $\rm Q^3$ has the form

$$V_{\rm 3N}^{\rm Q^3} = V_{c_E}^{(l)} + V_{c_E}^{(t)} + V_{c_E}^{(d)} + V_{c_D}^{(d)} + V_{\rm 3N}^{\rm (TPE)}\,, \tag{S15}$$

where

$$V_{c_E}^{(d)} = \frac{1}{6}\frac{c_E^{(d)}}{2f_\pi^4\Lambda_\chi} : \sum_{\vec{n}} \left[\rho^{(d)}(\vec{n})\right]^3 :\,, \tag{S16}$$

$$V_{c_D}^{(d)} = -\frac{c_D^{(d)}\,g_A}{4f_\pi^4\Lambda_\chi} \sum_{\vec{n},S,I}\sum_{\vec{n}',S'} : \rho^{(0)}_{S',I}(\vec{n}')f_{S',S}(\vec{n}'-\vec{n})\rho^{(d)}_{S,I}(\vec{n})\rho^{(d)}(\vec{n}) :\,, \tag{S17}$$

$$V_{c_E}^{(l)} = c_E^{(l)} \sum_{\vec{n},\vec{n}',\vec{n}''} \rho^{(0)}(\vec{n})\,\rho^{(0)}(\vec{n}')\,\rho^{(0)}(\vec{n}'')\delta_{|\vec{n}-\vec{n}'|,1}\,\delta_{|\vec{n}-\vec{n}''|,1}\,\delta_{|\vec{n}'-\vec{n}''|,2}, \tag{S18}$$

$$V_{c_E}^{(t)} = c_E^{(t)} \sum_{\vec{n},\vec{n}',\vec{n}''} \rho^{(0)}(\vec{n})\,\rho^{(0)}(\vec{n}')\,\rho^{(0)}(\vec{n}'')\delta_{|\vec{n}-\vec{n}'|,\sqrt{2}}\,\delta_{|\vec{n}-\vec{n}''|,\sqrt{2}}\,\delta_{|\vec{n}'-\vec{n}''|,\sqrt{2}}. \tag{S19}$$

The $V_{\rm 3N}^{\rm (TPE)}$ potential can be separated into the following three parts,

$$\begin{aligned} V_{\rm 3N}^{\rm (TPE1)} = & \frac{c_3}{f_\pi^2}\frac{g_A^2}{4f_\pi^2}\sum_{S,S',S'',I}\sum_{\vec{n},\vec{n}',\vec{n}''} \\ & \times : \rho^{(0)}_{S',I}(\vec{n}')\, f_{S',S}(\vec{n}'-\vec{n}) f_{S'',S}(\vec{n}''-\vec{n})\rho^{(0)}_{S'',I}(\vec{n}'')\,\rho^{(0)}(\vec{n}) : \end{aligned} \tag{S20}$$

$$\begin{aligned} V_{\rm 3N}^{\rm (TPE2)} = & -\frac{2c_1}{f_\pi^2}\frac{g_A^2 M_\pi^2}{4f_\pi^2}\sum_{S,S',I}\sum_{\vec{n},\vec{n}',\vec{n}''} \\ & \times : \rho^{(0)}_{S',I}(\vec{n}')\, f^{\pi\pi}_{S'}(\vec{n}'-\vec{n}) f^{\pi\pi}_{S}(\vec{n}''-\vec{n})\rho^{(0)}_{S,I}(\vec{n}'')\,\rho^{(0)}(\vec{n}) : , \end{aligned} \tag{S21}$$

$$\begin{aligned} V_{\rm 3N}^{\rm (TPE3)} = & \frac{c_4}{2f_\pi^2}\left(\frac{g_A}{2f_\pi}\right)^2 \sum_{S_1,S_2,S_3}\sum_{I_1,I_2,I_3}\sum_{S',S''}\sum_{\vec{n},\vec{n}',\vec{n}''} \varepsilon_{S_1,S_2,S_3}\varepsilon_{I_1,I_2,I_3} \\ & \times : \rho^{(0)}_{S',I_1}(\vec{n}')\, f_{S',S_1}(\vec{n}'-\vec{n}) f_{S'',S_2}(\vec{n}''-\vec{n})\rho^{(0)}_{S'',I_2}(\vec{n}'')\,\rho^{(0)}_{S_3,I_3}(\vec{n}) : . \end{aligned} \tag{S22}$$

We perform our calculations using lattice spacing $a = 1.32$ fm, and we determine the low-energy constants (LECs) of the 2N short-range interaction up to N3LO of χEFT by reproducing the neutron-proton scattering phase shifts and mixing angles of the Nijmegen partial wave analysis (PWA).[25] The lattice spacing of $a = 1.32$ fm corresponds to the momentum space cutoff of 470 MeV, which corresponds to the resolution scale at which the hidden spin-isospin symmetry of the NN interactions is best fulfilled.[26] In Fig. S1 we plot the calculated neutron-proton scattering phase shifts and mixing angles up to N3LO of χEFT as functions of relative momenta with comparison to the Nijmegen PWA. Only the statistical errors and not systematic errors are included in the Nijmegen PWA. In the next section we discuss the approach used to estimate the uncertainties in our calculations and the determination of the LECs of the three-nucleon interactions.

S3 Hamiltonian Translators

Before describing how wavefunction matching is implemented in practice, we first discuss a class of transformations called Hamiltonian translators. Let H_A and H_B be two Hamiltonians acting on the same linear space. H_B corresponds to the simple Hamiltonian that we called H^S in the main text and H_A corresponds to the high-fidelity Hamiltonian H. We have temporarily changed the notation here so that we can use a convenient notation with A and B as subscripts. Suppose that U_{AB} is a unitary transformation mapping all the eigenvectors of H_B to all the eigenvectors of H_A. We then call U_{AB} a Hamiltonian translator from H_B to H_A. Clearly, $U_{BA} = U_{AB}^\dagger$ is then a Hamiltonian translator from H_A to H_B. We note the curious fact that $H_A' = U_{BA}H_AU_{AB}$ is a Hamiltonian with energy eigenvalues identical to those of H_A, but with eigenvectors identical to those of H_B. Similarly, $H_B' = U_{AB}H_BU_{BA}$ is a Hamiltonian with energy eigenvalues identical to those of H_B, but with eigenvectors identical to those of H_A.

Since H_A' and H_B share the same eigenvectors, H_A' and H_B commute with each other. In order to compute any energy eigenvalue of H_A', it suffices to prepare the corresponding eigenvector of H_B and compute the energy expectation value of H_A'. We can express these facts using the language of perturbation theory. If we write $H_A' = H_B + (H_A' - H_B)$, then the zeroth-order expansion of the eigenvectors is exact, and the first-order expansion of the energies is exact.

We can construct Hamiltonian translators using quantum adiabatic evolution.[27] Let f be a smooth function such that $f(0) = 0$ and $f(1) = 1$. Then for any $T > 0$, we can define the time-dependent Hamiltonian $H_T(t) = f(\frac{t}{T})H_A + [1 - f(\frac{t}{T})]H_B$. We also define the unitary transformation

$$U_T = \overleftarrow{T}\exp\left[-i\int_0^T H_T(t)dt\right], \tag{S23}$$

where $\overleftarrow{T}$ is the time ordering symbol placing operators at later times on the left. In the limit of large T, U_T is a Hamiltonian translator from H_B to H_A. In the limit of large T, U_T maps every eigenvector of H_B to an eigenvector of H_A. Within each symmetry subspace that is invariant under both Hamiltonians H_A and H_B, the mapping U_T preserves the ordering of energy eigenvalues.

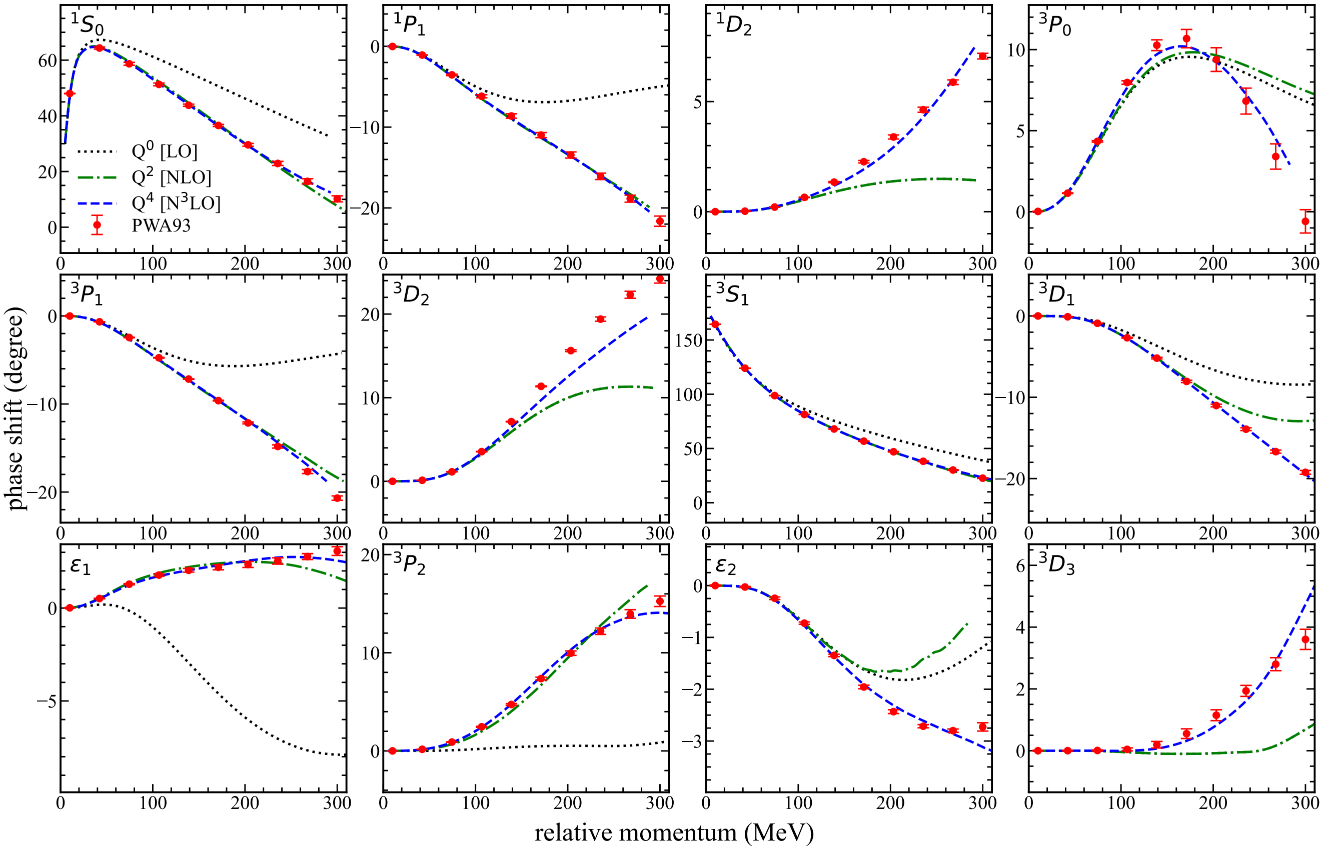

Figure S1. Neutron-proton scattering phase shifts and mixing angles. Plots of the neutron-proton scattering phase shifts and mixing angles as functions of relative momenta with lattice spacing $a = 1.32$ fm. For comparison we also plot the phase shifts and mixing angles extracted from the Nijmegen partial wave analysis.[25]

S4 Wavefunction Matching

As discussed in the main text, we let H^S be a simple Hamiltonian that is easily computable and H be a Hamiltonian with realistic interactions. Wavefunction matching can be viewed as an approximate Hamiltonian translator from H^S to H. It is only an approximate translator because the unitary transformation U will be restricted to the space of two nucleons up to some maximum separation distance R and will only map the lowest eigenvector of H^S to the lowest eigenvector of H in each scattering channel.

In the following, we restrict our focus to the space of two nucleons in some angular-momentum scattering channel. We impose hard wall boundary conditions at some very large separation distance R_{wall}. The energy eigenstates of H for our chosen two-nucleon scattering channel will be denoted $|\psi_n\rangle$ for $n = 0, 1, \cdots$, and E_n will be the corresponding energy eigenvalues. We let the corresponding energy eigenstates for H^S be denoted $|\psi_n^S\rangle$. Here we describe the implementation of wavefunction matching in lattice calculations. The implementation of wavefunction matching in continuous space is detailed in Section S10.

We define a finite-range projection operator P_R that projects out the portion of the two-nucleon state with separation distance less than or equal to R. We let $|m\rangle$ for $m = 1, \cdots, m_R$ be an orthogonal basis spanning the set of two-nucleon channel states up to that finite range so that $P_R = \sum_{m=1}^{m_R} |m\rangle\langle m|$. In the lattice calculations presented here, we take $|m\rangle$ to be radial position eigenstates, sorted according to increasing radial distance. See, for example, Ref.[28] for a discussion of radial position eigenstates on the lattice. For the calculations described in the main text, the chosen value for R is 3.72 fm. The dependence of the results upon R is discussed in some detail over the next few sections.

Let $|\psi_0\rangle_R$ and $|\psi_0^S\rangle_R$ be the normalized finite-range portions of the ground state wavefunctions for H and H^S respectively,

$$|\psi_0\rangle_R = \frac{P_R\,|\psi_0\rangle}{\|P_R\,|\psi_0\rangle\|}, \quad |\psi_0^S\rangle_R = \frac{P_R\,|\psi_0^S\rangle}{\|P_R\,|\psi_0^S\rangle\|}. \tag{S24}$$

Let us define a unitary transformation U such that $|\psi_0^S\rangle_R$ is mapped to $|\psi_0\rangle_R$. There are several ways to define the remaining

properties of U. In this work, we use Gram-Schmidt orthogonalization to define an ordered sequence of orthonormal basis states, $\{|\psi_0\rangle_R, |1\rangle_\perp, \cdots, |m_R - 1\rangle_\perp\}$ and, similarly, $\{|\psi_0^S\rangle_R, |1\rangle_\perp^S, \cdots, |m_R - 1\rangle_\perp^S\}$. We require that U maps each basis vector $|j\rangle_\perp^S$ to the corresponding basis vector $|j\rangle_\perp$ for each $j = 1, \cdots, m_R - 1$.

Another choice for the unitary transformation U is to use a Householder reflection.[29] This is implemented by defining the normalized difference vector

$$|\psi_0^\Delta\rangle_R = \frac{|\psi_0\rangle_R - |\psi_0^S\rangle_R}{\| |\psi_0\rangle_R - |\psi_0^S\rangle_R \|}, \tag{S25}$$

and defining the unitary transformation as

$$U = 1 - 2\,_R|\psi_0^\Delta\rangle\,\langle\psi_0^\Delta|_R. \tag{S26}$$

It is straightforward to verify that U maps $|\psi_0^S\rangle_R$ to $|\psi_0\rangle_R$. While the Householder reflection is simple and elegant, it has the disadvantage that U is not well defined in the limit that H^S approaches H.

In order to fix the problem, we can instead use a Givens rotation to rotate vectors in the two-dimensional subspace spanned by $|\psi_0^S\rangle_R$ and $|\psi_0\rangle_R$.[30] This can be implemented as a product of two Householder reflections. Let $|\psi_0^{S,\perp}\rangle_R$ be a linear combination of $|\psi_0^S\rangle_R$ and $|\psi_0\rangle_R$ that is normalized and perpendicular to $|\psi_0^S\rangle_R$. This can be written explicitly as

$$|\psi_0^{S,\perp}\rangle_R = \frac{|\psi_0\rangle_R - {}_R\langle\psi_0^S|\psi_0\rangle_R\,|\psi_0^S\rangle_R}{\| |\psi_0\rangle_R - {}_R\langle\psi_0^S|\psi_0\rangle_R\,|\psi_0^S\rangle_R \|}. \tag{S27}$$

We can check that $|\psi_0^{S,\perp}\rangle_R$ is a normalized linear combination of $|\psi_0^S\rangle_R$ and $|\psi_0\rangle_R$ that is perpendicular to $|\psi_0^S\rangle_R$. The unitary transformation for Givens rotation is

$$U = U^\Delta U^{S,\perp}, \tag{S28}$$

where

$$U^{S,\perp} = 1 - 2\,_R|\psi_0^{S,\perp}\rangle\,\langle\psi_0^{S,\perp}|_R, \tag{S29}$$

and

$$U^\Delta = 1 - 2\,_R|\psi_0^\Delta\rangle\,\langle\psi_0^\Delta|_R. \tag{S30}$$

It is straightforward to verify that U maps $|\psi_0^S\rangle_R$ to $|\psi_0\rangle_R$.

Once the unitary transformation U is determined, we proceed by defining the transformed Hamiltonian $H' = U^\dagger H U$. Let $|\psi_0'\rangle$ be the ground state of H'. In the following, we ignore any irrelevant overall phases in the definition of $|\psi_0'\rangle$. We note that $|\psi_0'\rangle = U^\dagger |\psi_0\rangle$. The finite range part of $|\psi_0'\rangle$ is

$$|\psi_0'\rangle_R = \frac{P_R|\psi_0'\rangle}{\|P_R|\psi_0'\rangle\|}. \tag{S31}$$

Since U is trivial at distances greater than R, $|\psi_0'\rangle$ must equal $|\psi_0\rangle$ at distances greater than R. At distances less than R, $|\psi_0'\rangle_R$ must equal $|\psi_0^S\rangle_R$. Hence, the finite range behavior of $|\psi_0'\rangle$ must be proportional to $|\psi_0^S\rangle$. Let the constant of proportionality be denoted κ,

$$\psi_0'(r) = \kappa\psi_0^S(r) \text{ for } r < R. \tag{S32}$$

If $|\psi_0\rangle$ and $|\psi_0^S\rangle$ have approximately equal normalizations at distances greater than R, then the constant proportionality will be numerically close to 1.

In order for wavefunction matching to accelerate the convergence of perturbation theory, it is important that E_0 and E_S are close in energy and the asymptotic normalization ratio κ is close to 1. In the ideal case where $E_0 = E_S$ and $\kappa = 1$, $|\psi_0'\rangle$ and $|\psi_0^S\rangle$ will be identical. In that case, first-order perturbation theory for the ground state energy is exact and the perturbative corrections to the ground state wavefunction vanish. For the general case where E_0 and E_S are close in energy and the asymptotic normalization ratio κ is approximately 1, then $|\psi_0'\rangle$ and $|\psi_0^S\rangle$ are nearly equal. This accelerates the convergence of perturbation theory for the ground state. Since the other low-energy wavefunctions have the same finite-range behavior as the ground state, this is enough to accelerate the convergence of perturbation theory for all the low-energy states of H' when starting from eigenstates of H^S.

For the many-body calculations presented in the main text, we perform the following steps. We first write the transformed two-nucleon Hamiltonian H' as $H' = H^S + (H' - H^S)$. We then prepare eigenstates of H^S and apply corrections up to first order in perturbation theory to get the properties of the eigenstates of H'. The three-nucleon interactions are added to H' at this stage. In order to accelerate the convergence of perturbation theory further, we consider the more general partition $H' = H'^S + (H' - H'^S)$. The modified simple Hamiltonian H'^S has the same form as H^S in Eq. (S1), but we allow for different coupling strengths $c_{\mathrm{SU}(4)}$ and c_I. We then minimize the energy to optimize the parameters. We should clarify that the parameters $c_{\mathrm{SU}(4)}$ and c_I in H'^S are only used to improve the quality of the variational trial states and have nothing to do with the actual interaction parameters of the Hamiltonian.

S5 Analyticity of Wavefunction Matching

The unitary transformation used in wavefunction matching is locally integrable and differs from the identity only within a compact domain. Let $\mathbf{S}, \mathbf{S}'$ represent spin indices and $\mathbf{I}, \mathbf{I}'$ represent isospin indices. The nontrivial part of the wavefunction matching unitary transformation is

$$f(\mathbf{S},\mathbf{S}';\mathbf{I},\mathbf{I}';\mathbf{r},\mathbf{r}') \equiv U(\mathbf{S},\mathbf{S}';\mathbf{I},\mathbf{I}';\mathbf{r},\mathbf{r}') - \delta_{\mathbf{S},\mathbf{S}'}\delta_{\mathbf{I},\mathbf{I}'}\delta^3(\mathbf{r}-\mathbf{r}'), \tag{S33}$$

where

$$f(\mathbf{S},\mathbf{S}';\mathbf{I},\mathbf{I}';\mathbf{r},\mathbf{r}') = 0 \text{ if } |\mathbf{r}| > R \text{ or } |\mathbf{r}'| > R. \tag{S34}$$

By assumption, we have regulated all divergences in our interactions so that $f(\mathbf{S},\mathbf{S}';\mathbf{I},\mathbf{I}';\mathbf{r},\mathbf{r}')$ is finite everywhere. In momentum space, the nontrivial part is then

$$\tilde{f}(\mathbf{S},\mathbf{S}';\mathbf{I},\mathbf{I}';\mathbf{p},\mathbf{p}') = \int d^3\mathbf{r}d^3\mathbf{r}' e^{i\mathbf{p}\cdot\mathbf{r}}e^{i\mathbf{p}'\cdot\mathbf{r}'} f(\mathbf{S},\mathbf{S}';\mathbf{I},\mathbf{I}';\mathbf{r},\mathbf{r}'). \tag{S35}$$

Since we are integrating a finite-valued function over a compact region, we can differentiate with respect to the momentum variables $\mathbf{p}$ and $\mathbf{p}'$,

$$\nabla_{\mathbf{p}}\tilde{f}(\mathbf{S},\mathbf{S}';\mathbf{I},\mathbf{I}';\mathbf{p},\mathbf{p}') = \int d^3\mathbf{r}d^3\mathbf{r}'\ i\mathbf{r}\ e^{i\mathbf{p}\cdot\mathbf{r}}e^{i\mathbf{p}'\cdot\mathbf{r}'} f(\mathbf{S},\mathbf{S}';\mathbf{I},\mathbf{I}';\mathbf{r},\mathbf{r}'), \tag{S36}$$

$$\nabla_{\mathbf{p}'}\tilde{f}(\mathbf{S},\mathbf{S}';\mathbf{I},\mathbf{I}';\mathbf{p},\mathbf{p}') = \int d^3\mathbf{r}d^3\mathbf{r}'\ i\mathbf{r}'\ e^{i\mathbf{p}\cdot\mathbf{r}}e^{i\mathbf{p}'\cdot\mathbf{r}'} f(\mathbf{S},\mathbf{S}';\mathbf{I},\mathbf{I}';\mathbf{r},\mathbf{r}'). \tag{S37}$$

We conclude that the wavefunction matching transformation is analytic everywhere in momentum space. It does not produce any new non-analytic behavior. It defines a new low-energy effective field theory with the same breakdown scale as the original low-energy effective field theory. This means that the effective field theory corrections for H and H' will have the same asymptotic scaling at high orders.

S6 Dependence on the Wavefunction Matching Radius R

For each scattering channel where wavefunction matching is used, we assume that E_0 and E_S are close in energy and the asymptotic normalization ratio κ is close to 1. These conditions are required for wavefunction matching to accelerate perturbation theory. They are satisfied for the applications of wavefunction matching to two-nucleon interactions in the main text, and they are also satisfied for the examples we discuss in the next few sections.

Let r_Δ be the largest radial distance for which the interactions comprising H and H^S are different and the difference is significant in magnitude. For the two-nucleon interactions in the main text, r_Δ is approximately one lattice spacing, or 1.32 fm. For the higher partial waves, the interactions vanish at zero distance and r_Δ extends somewhat further to $\sqrt{2}$ times the lattice spacing, or 1.86 fm. Since $E_0 \approx E_S$ and $\kappa \approx 1$, it follows that $\psi_0(r) \approx \psi_0^S(r)$ for all r greater than r_Δ.

The unitary transformation U used in wavefunction matching maps $|\psi_0^S\rangle_R$ to $|\psi_0\rangle_R$. Since the wavefunctions $|\psi_0^S\rangle_R$ and $|\psi_0\rangle_R$ are nearly equal for $r \geq r_\Delta$, the action of U on $|\psi_0^S\rangle_R$ can therefore be truncated to radial distances $r < r_\Delta$. For the transformation of $|\psi_0^S\rangle_R$, we conclude that there is no dependence on R once R is greater than r_Δ. On the other hand, U also transforms other two-body states at distances $r < R$. However, these states are orthogonal to $|\psi_0^S\rangle_R$. These states must have an extra node between $r = 0$ and $r = R$ and therefore correspond to a relative momentum of size $O(2\pi/R)$ or larger. The dependence on R can only be seen in the interactions of the high-energy modes with momenta above $2\pi/R$. The low-energy physics of wavefunction matching is independent of R. While there are interactions in H' that reach up to radial distances of R, these interactions only couple to high-energy modes. This analysis shows that wavefunction matching is very different from renormalization group evolution. Changing R does not change the low-energy resolution scale, and H' behaves as a two-body Hamiltonian with interaction range set by r_Δ. This is in addition to any long-range interaction features such as the one-pion exchange potential common to both H and H^S and therefore trivially carried over to H'.

The value of $R = 3.72$ fm used in the main text corresponds to a momentum scale of $2\pi/R = 333$ MeV. Since 333 MeV is a high-momentum scale comparable to the cutoff scale of our low-energy effective field theory, we expect the low-energy physics is largely independent of R in this range. This is in fact what is observed. In Fig. S2 we plot the energies of ^{3}H and ^{4}He for different values of the wavefunction matching radius R. We show lattice results at LO, NLO and N3LO in chiral effective field theory for all possible values of R less than or equal to 3.72 fm using two-nucleon interactions only. The corrections are calculated using first-order perturbation theory. The gray band is the predicted result from Ref.,[31] and the black open box shows the empirical point. We see that the results for $R > 2.7$ fm are largely independent of R. The deviation from the Tjon band for $R < 2.7$ fm is likely due to perturbation theory corrections beyond first order. The additional deviation for the LO results is due to the fact that Coulomb is not included. The fast convergence of perturbation theory in wavefunction matching requires that

the radius R is somewhat larger than r_Δ. This appears to be satisfied for $R = 2.94$ fm, 3.22 fm and 3.72 fm, and the results are nearly identical for the values for $R > 2.7$ fm. Also, the condition for the fast convergence of perturbation theory is consistently met at LO, NLO, and N3LO in chiral effective field theory as seen in Fig. S2.

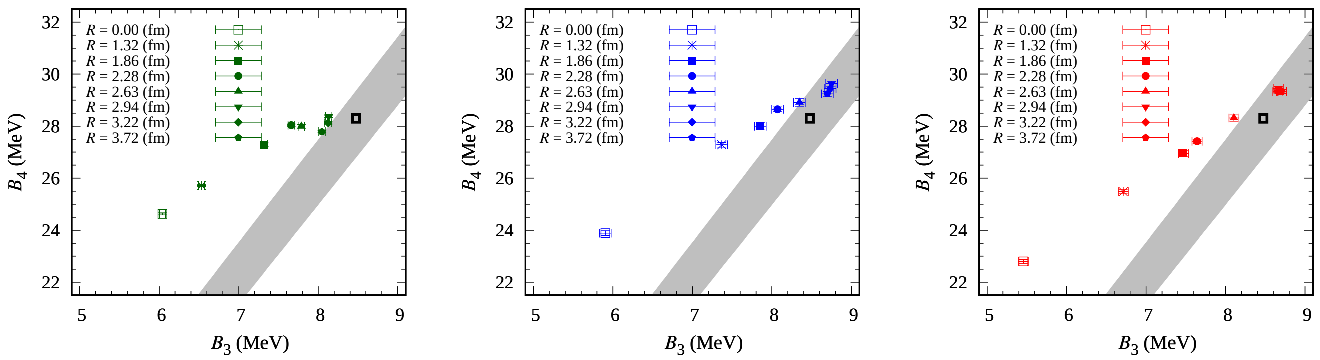

Figure S2. Binding energies of ^{3}H and ^{4}He for different values of the wavefunction matching radius R. We show lattice results at LO, NLO and N3LO in chiral effective field theory for $R = 0.0$ fm, 1.32 fm, 1.86 fm, 2.28 fm, 2.63 fm, 2.94 fm, 3.22 fm, and 3.72 fm. The gray band is the predicted result from Ref.,[31] and the black open box shows the empirical point.

To further elucidate the above discussion, we assert that the slow convergence of perturbation theory not only causes deviations from the Tjon line but also disrupts the convergence of chiral effective field theory. This observation is exemplified by the binding energies of ^{2}H, ^{3}H, and ^{4}He presented in Table S1 for varying values of R in the range of 0 fm to 3.72 fm using two-nucleon interactions only. The outcomes clearly illustrate that when first-order perturbation theory inadequately approximates the solution, the convergence of chiral effective field theory becomes less controlled. Nevertheless, a remarkable resolution to this challenge emerges, as demonstrated by the results in Table S1. The rapid convergence of perturbation theory through the use of wavefunction matching elegantly rectifies the problem.

Table S1. Binding energies of ^{2}H, ^{3}H and ^{4}He for different values of the wavefunction matching radius R. We show lattice results at LO, NLO and N3LO in chiral effective field theory for $R = 0.0$ fm, 1.32 fm, 1.86 fm, 2.28 fm, 2.63 fm, 2.94 fm, 3.22 fm, and 3.72 fm.

Nuclei	R (fm)							
	0.00	1.32	1.86	2.28	2.63	2.94	3.22	3.72
$B_{^2\mathrm{H,LO}}$	0.449	0.669	0.998	1.140	1.231	1.502	1.630	1.650
$B_{^2\mathrm{H,NLO}}$	0.345	0.893	1.247	1.295	1.517	1.853	1.916	1.977
$B_{^2\mathrm{H,N3LO}}$	0.621	1.091	1.341	1.372	1.584	1.897	1.953	2.01
$B_{^3\mathrm{H,LO}}$	6.04(4)	6.53(4)	7.32(4)	7.66(4)	7.79(4)	8.13(4)	8.12(4)	8.05(4)
$B_{^3\mathrm{H,NLO}}$	5.46(6)	6.71(6)	7.47(6)	7.64(6)	8.11(6)	8.66(6)	8.66(6)	8.70(7)
$B_{^3\mathrm{H,N3LO}}$	5.91(7)	7.37(7)	7.85(7)	8.07(7)	8.35(7)	8.75(7)	8.73(7)	8.70(8)
$B_{^4\mathrm{He,LO}}$	24.63(4)	25.72(4)	27.29(7)	28.04(4)	27.99(7)	28.37(5)	28.12(5)	27.79(5)
$B_{^4\mathrm{He,NLO}}$	22.80(8)	25.48(8)	26.95(12)	27.42(8)	28.31(12)	29.45(8)	29.32(8)	29.33(8)
$B_{^4\mathrm{He,N3LO}}$	23.88(9)	27.28(9)	28.00(14)	28.65(9)	28.91(15)	29.63(9)	29.44(9)	29.24(9)

S7 Induced Three-Body Interactions in Wavefunction Matching: Born-Oppenheimer Analysis

In this section, we study the induced three-body interactions generated by wavefunction matching and the dependence on the wavefunction matching radius R. For this purpose, we use a Born-Oppenheimer analysis similar to the approaches used in Ref.[32,33] Using a three-dimensional lattice, we consider two scalar particles with infinite mass and a third scalar particle with finite mass, $m = 939$ MeV. For notational convenience, we call the two particles with infinite mass A and B and the particle with finite mass C.

We take the lattice spacing to be 1.32 fm. For the kinetic energy operator for particle C, we take the nearest-neighbor lattice

action,

$$K_C = \frac{1}{2m}\sum_{\vec{n}}\sum_{l=1,2,3}[a_C^\dagger(\vec{n}+\hat{l}) - a_C^\dagger(\vec{n})][a_C(\vec{n}+\hat{l}) - a_C(\vec{n})]. \tag{S38}$$

We consider the Hamiltonian H,

$$\begin{aligned} H = K_C + &\sum_{\vec{n}} a_A^\dagger(\vec{n})a_A(\vec{n})[c_{AC}a_C^\dagger(\vec{n})a_C(\vec{n}) + c'_{AC}\tilde{a}_C^\dagger(\vec{n})\tilde{a}_C(\vec{n})] \\ &+ \sum_{\vec{n}} a_B^\dagger(\vec{n})a_B(\vec{n})[c_{BC}a_C^\dagger(\vec{n})a_C(\vec{n}) + c'_{BC}\tilde{a}_C^\dagger(\vec{n})\tilde{a}_C(\vec{n})], \end{aligned} \tag{S39}$$

where the nonlocally-smeared annihilation and creation operators are

$$\tilde{a}_C(\vec{n}) = a_C(\vec{n}) + s_{\rm NL}\sum_{|\vec{n}'-\vec{n}|=1} a_C(\vec{n}'). \tag{S40}$$

In dimensionless lattice units, we use the parameter values $s_{\rm NL} = 0.2$, $c_{AC} = c_{BC} = 1.0$, and $c'_{AC} = c'_{BC} = -0.5$. When one of the infinite mass particles is removed, the ground state of the remaining system is a bound dimer with energy -2.864 MeV. For this example, the interactions are nonzero only in the S-wave channel.

For the simple Hamiltonian, H^S, we remove the short-range repulsive interactions and use the parameter values $s_{\rm NL} = 0.2$, $c_{AC} = c_{BC} = 0.0$, and $c'_{AC} = c'_{BC} = -0.18$. With this choice of parameters, the two Hamiltonians, H and H_s are well matched, meaning that the two-body S-wave ground state energies are nearly the same and the ratio of asymptotic normalization coefficients κ is close to 1. We now apply wavefunction matching to H and H^S and produce a new Hamiltonian H' that is unitarily equivalent to H at the two-body level. Wavefunction matching is performed on the S-wave interactions between A and C and the S-wave interactions between B and C. We vary the range R for the wavefunction matching transformation from 1.5 to 5.5 lattice units. We use the Gram-Schmidt wavefunction matching procedure described in Section S4.

For comparison, we also consider a general unitary transformation U of the S-wave interactions between A and C and the S-wave interactions between B and C. Let r and r' denote the incoming and outgoing radial separations between the particles, respectively. We allow r and r' to range from 0 to R, and define $U = \exp[i(M+M^\dagger)/2]$, where each matrix element $M(r,r')$ is a uniformly-distributed random real number between -1 and $+1$.

In Fig. S3 we show the three-body ground state energies for H and H' versus separation distance r for transformation radius 1.5 lattice units or 2.0 fm. r is the distance between the infinite mass particles A and B. The left panel shows the random unitary matrix results and the right panel shows the wavefunction matching results. The difference between the three-body ground state energies for H and H' gives a measure of the size of the induced three-body interactions versus separation distance r. We see that the results are qualitatively similar for the two cases. The induced three-body interaction is small in magnitude overall and is negligible at distances greater than 2 lattice units.

In Fig. S4, we show the three-body ground state energies for H and H' versus separation distance r for transformation radius 2.5 lattice units or 3.3 fm. Fig. S5 corresponds to 3.5 lattice units or 4.6 fm, Fig. S6 corresponds to 4.5 lattice units or 5.9 fm, and Fig. S7 corresponds to 5.5 lattice units or 7.2 fm. We see that the trends for the random unitary and wavefunction matching are very different with increasing radius R. The induced three-body interaction for the random unitary grows in magnitude and range with increasing R. The induced three-body interaction for the wavefunction matching remains small and does not grow with R.

The reason that the induced three-body interaction remains small in magnitude comes from the fact that H, H^S, H' all have non-singular interactions with a short range. We can use naive dimensional analysis to predict that the relative strength of the induced three-body interaction in d spatial dimensions is suppressed by $(pr)^d$, where p is the typical particle momentum and r is the interaction range.

S8 Induced Three-Body Interactions in Wavefunction Matching: Three Particles in One Dimension

Let us consider three distinguishable spinless particles with the same mass, $m = 939$ MeV, in one spatial dimension. We again take the lattice spacing to be 1.32 fm. For the kinetic energy operator, we use the order $O(a^4)$-improved lattice action,

$$K = -\frac{1}{2m}\sum_{n}\sum_{k=1,2,3} a_k^\dagger(n)\left[\frac{1}{90}a_k(n+3) - \frac{3}{20}a_k(n+2) + \frac{3}{2}a_k(n+1) - \frac{49}{18}a_k(n) + \frac{3}{2}a_k(n-1) - \frac{3}{20}a_k(n-2) + \frac{1}{90}a_k(n-3)\right]. \tag{S41}$$

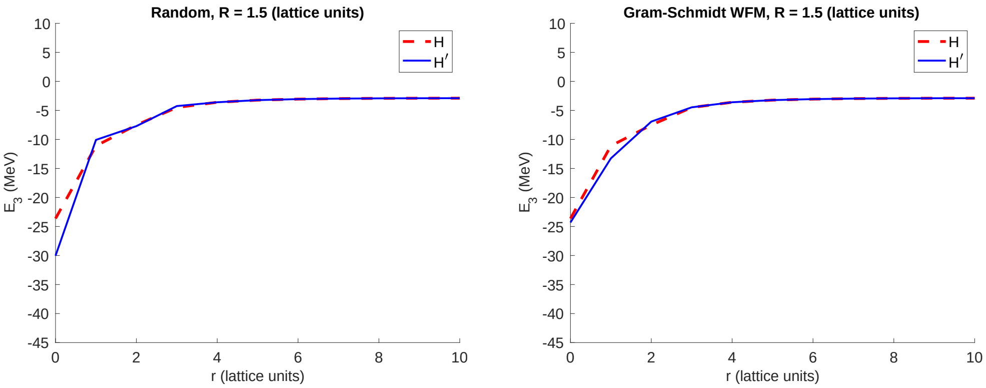

Figure S3. Three-body ground state energies for H and H' versus separation distance for transformation radius 1.5 lattice units or 2.0 fm. Left Panel: Results for the random unitary matrix. **Right Panel:** Results for wavefunction matching.

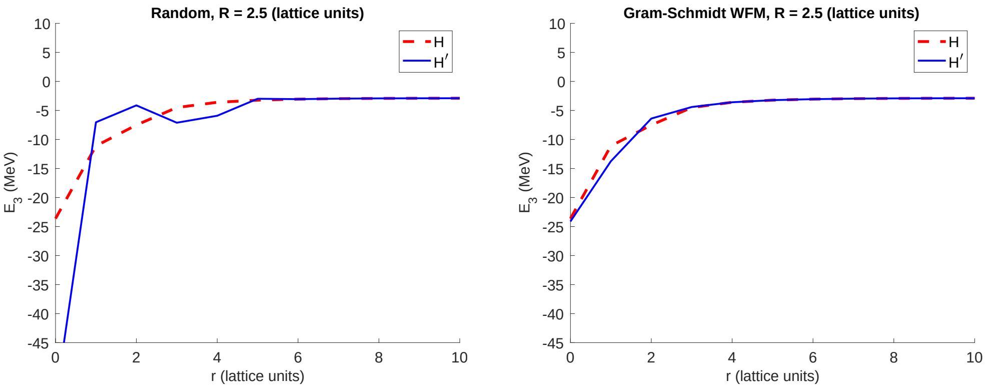

Figure S4. Three-body ground state energies for H and H' versus separation distance for transformation radius 2.5 lattice units or 3.3 fm. Left Panel: Results for the random unitary matrix. **Right Panel:** Results for wavefunction matching.

We consider the Hamiltonian H,

$$H = K + c\sum_n \sum_{j<k} \rho_j(n)\rho_k(n) + c'\sum_n \sum_{j<k} \tilde{\rho}_j(n)\tilde{\rho}_k(n), \tag{S42}$$

where the point density $\rho_k(n)$ is

$$\rho_k(n) = a_k^\dagger(n)a_k(n) \tag{S43}$$

and the locally-smeared density is

$$\tilde{\rho}_k(n) = a_k^\dagger(n)a_k(n) + s_L \sum_{|n'-n|=1} a_k^\dagger(n')a_k(n'). \tag{S44}$$

In dimensionless lattice units, we use the parameter values $s_{\rm L} = 0.2$, $c = 0.3$, and $c' = -0.35$. For the simple Hamiltonian, H^S, we use the same general form and take $s_{\rm L} = 0.2$, $c = 0.0$, and $c' = -0.10$. These choices for H and H^S are well matched since the two-body ground state energies are nearly the same and the ratio of asymptotic normalization coefficients κ is close to 1.

For each even parity two-body channel, we perform wavefunction matching using the Givens rotation method at three different values for the radius.[30] We use $R = 2.0$ fm (1.5 lattice units), $R = 4.6$ fm (3.5 lattice units), and $R = 7.2$ fm (5.5 lattice units). In Table S2, we display the lowest three two-body energies with even parity in a periodic box of length $L = 26.3$ fm, or 20 lattice units. E_2^H corresponds to H, $E_2^{H'}$ corresponds to H', $E_{2,\rm pert}^{H'}$ corresponds to H' computed using first-order perturbation theory, and $E_{2,\rm pert}^{H}$ corresponds to H computed using first-order perturbation theory. For the H' data, we show results for $R = 2.0$ fm, $R = 4.6$ fm, and $R = 7.2$ fm. We note that $E_2^{H'}$ exactly matches E_2^H for all values of R. This is expected since the wavefunction matching transformation is an exact unitary transformation at the two-body level. We also see that wavefunction

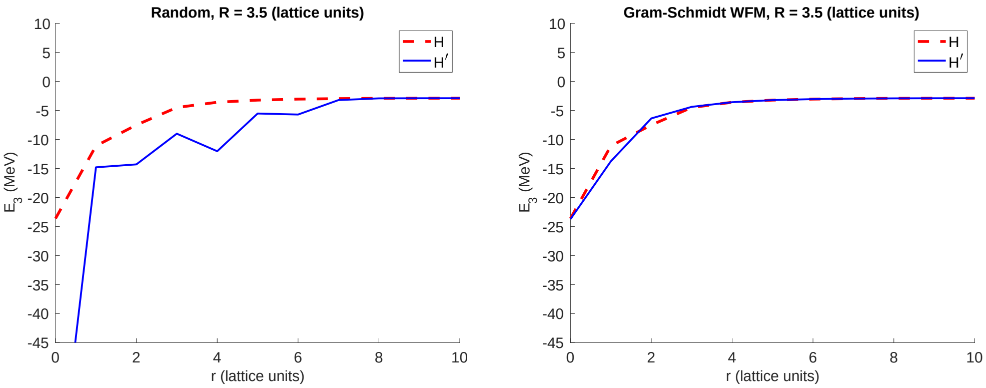

Figure S5. **Three-body ground state energies for H and H' versus separation distance for transformation radius 3.5 lattice units or 4.6 fm. Left Panel:** Results for the random unitary matrix. **Right Panel:** Results for wavefunction matching.

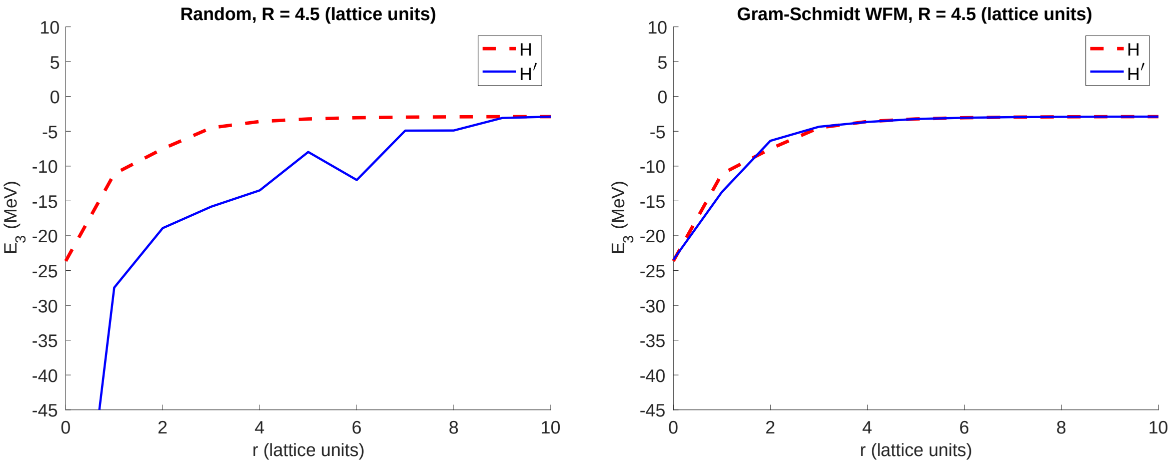

Figure S6. **Three-body ground state energies for H and H' versus separation distance for transformation radius 4.5 lattice units or 5.9 fm. Left Panel:** Results for the random unitary matrix. **Right Panel:** Results for wavefunction matching.

matching is significantly improving the accuracy of first-order perturbation theory for all values of R. $E^{H'}_{2,\text{pert}}$ is very close to $E^{H'}_2$ for each of the energy levels and for each value of R. This can be contrasted with $E^{H}_{2,\text{pert}}$, which has larger deviations from E^{H}_2.

In Table S3, the first two columns show the three-body energies for H and the transformed Hamiltonian H'. We compute the lowest six energy levels in a periodic box of length $L = 20$ lattice units. The second two columns show the corresponding results for the two-body energies using first-order perturbation theory, starting from eigenstates of H^S. We note that $E^{H'}_3$ is largely independent of the radius R for all six energy levels. This is in stark contrast with the behavior seen in similarity renormalization group flows, where the induced three-body interaction is very strongly dependent on the low-energy resolution scale. The range of the interactions of the transformed Hamiltonian H' does not increase with R and instead remains equal to the maximum of the ranges for H and H^S. The theoretical basis for this explained in Section S6.

Another interesting fact is that $E^{H'}_3$ is very close to E^{H}_3 for all six energy levels, meaning that the induced three-body interaction produced by wavefunction matching is numerically small. The reason was already explained in the previous section. It arises from the fact that H, H^S, H' all have non-singular interactions with a short range. The relative strength of the induced three-body interaction in d spatial dimensions is suppressed by $(pr)^d$, where p is the typical particle momentum and r is the interaction range.

We also note that $E^{H'}_{3,\text{pert}}$ is very close to $E^{H'}_3$ for all six energy levels. This shows that wavefunction matching is improving the convergence perturbation theory for the three-body system. The level of improvement can be seen from the much smaller discrepancy between the values for $E^{H'}_{3,\text{pert}}$ and $E^{H'}_3$ as compared with $E^{H}_{3,\text{pert}}$ and E^{H}_3.

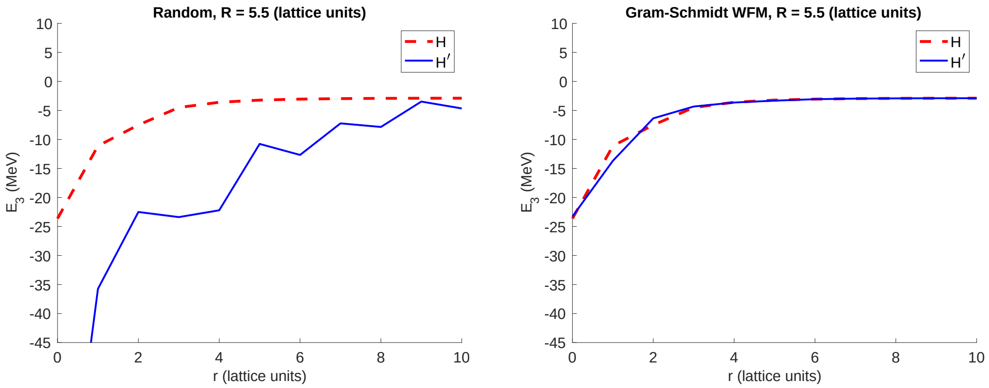

Figure S7. **Three-body ground state energies for H and H' versus separation distance for transformation radius 5.5 lattice units or 7.2 fm. Left Panel:** Results for the random unitary matrix. **Right Panel:** Results for wavefunction matching.

Table S2. **Lowest three two-body energies with even parity in a periodic box of length $L = 26.3$ fm.** From left to right, we present the energies for H (column 1), H' with $R = 2.0$ fm (column 2), H' with $R = 2.0$ fm using first-order perturbation theory (column 3), H' with $R = 4.6$ fm (column 4), H' with $R = 4.6$ fm using first-order perturbation theory (column 5), H' with $R = 7.2$ fm (column 6), H' with $R = 7.2$ fm using first-order perturbation theory (column 7), and H using first-order perturbation theory (column 8). All energies are units of MeV.

E_2^H	$E_2^{H'}$ $R = 2.0$ fm	$E_{2,\text{pert}}^{H'}$ $R = 2.0$ fm	$E_2^{H'}$ $R = 4.6$ fm	$E_{2,\text{pert}}^{H'}$ $R = 4.6$ fm	$E_2^{H'}$ $R = 7.2$ fm	$E_{2,\text{pert}}^{H'}$ $R = 7.2$ fm	$E_{2,\text{pert}}^{H}$
-4.0813	-4.0813	-4.0530	-4.0813	-4.0683	-4.0813	-4.0683	-3.7347
1.0448	1.0448	1.0404	1.0448	1.0408	1.0448	1.0497	1.0677
8.0314	8.0314	8.0438	8.0314	8.0635	8.0314	8.1024	8.1030

S9 Benchmark *Ab Initio* Calculations with and without Wavefunction Matching

In the following, we perform *ab initio* calculations of ^{3}H, ^{4}He, ^{8}Be and ^{12}C with and without wavefunction matching. We use the N2LO lattice interactions described in Ref.,[11] which was used to compute the energies and intrinsic structures of the low-lying states of ^{12}C. We use a spatial lattice spacing of $a = 1.64$ fm. The N2LO chiral interaction has two-nucleon interactions of the form

$$V_{2\text{N}} = V_{\text{SU}(4)} + V_{\text{OPE}} + V_{\text{contact}}^{(Q/\Lambda_\chi)^0} + V_{\text{contact}}^{(Q/\Lambda_\chi)^2}, \tag{S45}$$

and three-nucleon interactions of the form,

$$V_{3\text{N}} = V_{3N}^{\text{TPE}} + V_{3N}^{c_D} + V_{3N}^{c_E}. \tag{S46}$$

Details of this action can be found in Ref.[11] This interaction has much softer behavior at short distances than the high-fidelity chiral interactions used in the main text, and the phase shifts and mixing angles are fitted only up to 200 MeV relative momentum. This allows for fast convergence of perturbation theory without wavefunction matching, thus enabling benchmark comparisons of binding energies with and without wavefunction matching. Due to the softness of the N2LO interaction, the Tjon line relation connecting the ^{3}H and ^{4}He binding energies is not automatically satisfied using the nucleon-nucleon interactions alone. This has been noted in previous work[34] and is fixed by including significant smearing to the three-nucleon interactions $V_{3N}^{c_D}$ and $V_{3N}^{c_E}$. This allows for independent adjustment of the ^{3}H and ^{4}He binding energies.

For the wavefunction matching calculations, we use a simple two-nucleon Hamiltonian H^S with interactions of the form,

$$V_{2\text{N}}^S = V_{\text{SU}(4)}^S + V_{\text{OPE}} + V_{\text{contact}}^{S,(Q/\Lambda_\chi)^0}, \tag{S47}$$

which has the same features as the leading-order two-nucleon interaction in Eq. (S45) but with different parameters. The wavefunction radius used is $R = 3.29$ fm. For both cases, with and without wavefunction matching, we have included the three-nucleon interactions in Eq. (S46) and adjusted the coefficients of $V_{3N}^{c_D}$ and $V_{3N}^{c_E}$ to fit the empirical ground state energies for ^{3}H, ^{4}He, ^{8}Be and ^{12}C as well as possible.

In Table S4, we show the N2LO results obtained with and without wavefunction matching. We use spatial periodic boxes of lengths $L = 19.7$, 16.4, 16.4, and 13.1 fm for the calculations of ^{3}H, ^{4}He, ^{8}Be and ^{12}C, respectively, and we perform the

Table S3. Lowest six three-body energies in a periodic box of length $L = 26.3$ fm. From left to right, we present the energies for H (column 1), H' with $R = 2.0$ fm (column 2), H' with $R = 2.0$ fm using first-order perturbation theory (column 3), H' with $R = 4.6$ fm (column 4), H' with $R = 4.6$ fm using first-order perturbation theory (column 5), H' with $R = 7.2$ fm (column 6), H' with $R = 7.2$ fm using first-order perturbation theory (column 7), and H using first-order perturbation theory (column 8). All energies are units of MeV.

E_3^H	$E_3^{H'}$ $R = 2.0$ fm	$E_{3,\text{pert}}^{H'}$ $R = 2.0$ fm	$E_3^{H'}$ $R = 4.6$ fm	$E_{3,\text{pert}}^{H'}$ $R = 4.6$ fm	$E_3^{H'}$ $R = 7.2$ fm	$E_{3,\text{pert}}^{H'}$ $R = 7.2$ fm	$E_{3,\text{pert}}^{H}$
-14.5898	-14.7558	-14.6360	-14.7844	-14.7243	-14.6626	-14.5781	-13.0030
-4.3009	-4.2978	-4.0144	-4.2968	-4.0215	-4.3514	-4.0605	-3.6590
-4.3009	-4.2978	-3.9425	-4.2968	-3.9552	-4.3514	-4.0258	-3.5867
-4.0342	-4.0440	-3.9425	-4.0456	-3.9552	-4.1077	-4.0258	-3.5867
-3.1082	-3.1084	-3.0803	-3.1081	-3.0963	-3.0994	-3.0803	-2.7297
-3.1082	-3.1084	-3.0803	-3.1081	-3.0963	-3.0994	-3.0803	-2.7297

Euclidean time extrapolation to infinity. We present results for E_{2NFs} (two-nucleon interactions only), ΔE_{3NFs} (corrections from three-nucleon interactions) and $E_{\text{2NFs+3NFs}}$ (total). The excellent agreement for $E_{\text{2NFs+3NFs}}$ with and without wavefunction matching shows strong evidence that wavefunction matching is not changing the low-energy physics of these nuclei. This is a non-trivial check since the results at E_{2NFs} are different. Wavefunction matching is producing an induced three-nucleon interaction, but this induced three-nucleon interaction is consistent with a renormalization of the coefficients of the N2LO chiral three-nucleon interactions. Due to the softness of these interactions, there appears to be little impact of the wavefunction matching transformation beyond these induced three-nucleon interactions. We note that the two sets of calculations are performed with the same auxiliary field configurations, and so the computational errors for the two calculations are correlated.

Table S4. Binding energies of ^{3}H, ^{4}He, ^{8}Be and ^{12}C from the lattice action at N2LO[11] in chiral effective field theory. The first three columns show the results without wavefunction matching, and the last three columns are the results from calculations with wavefunction matching and R =3.29 fm.

Nuclei	Energies at N2LO w/o WFM (MeV)			Energies at N2LO w/ WFM (MeV)		
	E_{2NFs}	ΔE_{3NFs}	$E_{\text{2NFs+3NFs}}$	E'_{2NFs}	$\Delta E'_{\text{3NFs}}$	$E'_{\text{2NFs+3NFs}}$
^{3}H	-8.02(4)	-0.33(8)	-8.35(9)	-7.75(5)	-0.61(7)	-8.35(9)
^{4}He	-32.22(3)	4.57(7)	-27.65(8)	-29.50(3)	1.85(7)	-27.66(7)
^{8}Be	-64.34(8)	6.2(2)	-58.1(2)	-59.10(8)	1.0(1)	-58.1(2)
^{12}C	-86.98(4)	-5.2(1)	-92.2(1)	-80.30(4)	-11.92(9)	-92.2(1)

S10 Wavefunction Matching in Continuous Space

Wavefunction matching can be implemented in continuous space with only a slight modification from the lattice approach. There are several approaches that one can take, and we describe one straightforward implementation in this section. For each two-body scattering channel, we define a complete orthonormal basis $|v_j\rangle$ for the relative separation between the two particles. We require that each $|v_j\rangle$ is localized near $r = 0$, and one simple approach is to let $|v_j\rangle$ correspond to the radial eigenstates of some confining potential $V(r)$. Here, we consider the case where $V(r)$ is a harmonic oscillator potential.

We consider two particles in the center of mass frame. Both particles have mass m, and the orbital angular momentum L is arbitrary. We take the interaction between the two particles as

$$V(r) = \frac{1}{2}kr^2 = \frac{1}{2}\mu\Omega^2 r^2, \tag{S48}$$

where k is the spring constant, $\mu = m/2$ is the reduced mass, and $\Omega = \sqrt{k/\mu}$ is the oscillator frequency.

In order to perform the wavefunction matching unitary transformation, we first truncate the basis set to some finite subset $|v_j\rangle$, where j ranges from 1 to N. This truncation in N generates both an ultraviolet momentum regulator for high-energy excitations as well as an infrared spatial regulator for the radial extent of the wavefunctions. As N becomes large, both the ultraviolet momentum regulator and the infrared spatial regulator scale as the square root of N.[35–40]

For the calculations presented here, we take $m = 939$ MeV and $L = 0$. For the harmonic oscillator potential, we use $k = 10^8$ MeV^3, which corresponds to $\Omega = 462$ MeV. Calculations are carried out with a radial step size of 0.0197 fm and nearest-neighbor finite differences are used for calculations of the kinetic energy. In the left panel of Fig. S8, we show the four lowest harmonic oscillator basis states. In the right panel of Fig. S8, we plot the ground state wavefunctions for H, H^S, H' using $N = 4$ basis states.

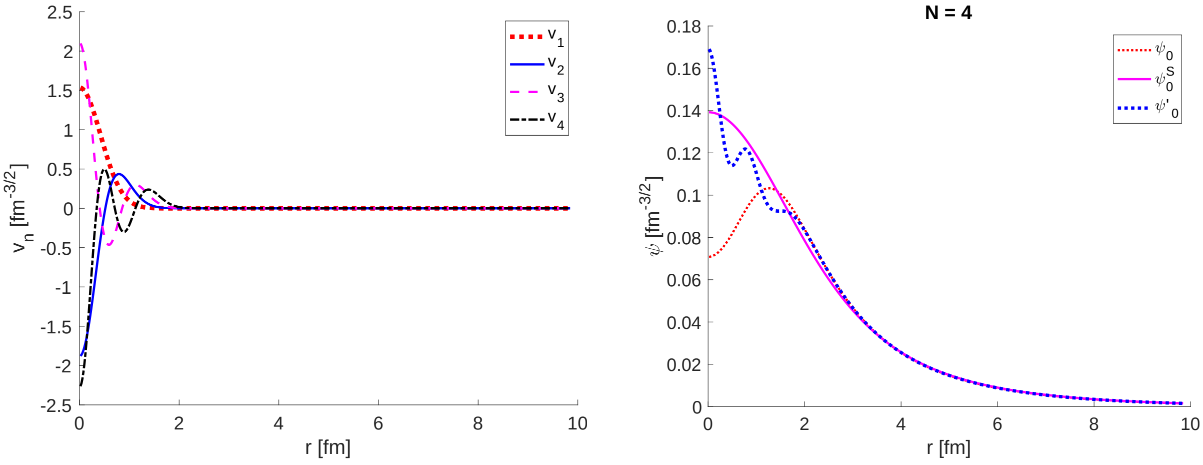

Figure S8. Basis states and ground state wavefunctions for continuum wavefunction matching. Left Panel: The four lowest harmonic oscillator basis states. **Right Panel:** Ground state wavefunctions for H, H^S, H' using $N = 4$ basis states.

In the left panel of Fig. S9, we plot the ground state wavefunctions using $N = 8$ basis states. In the right panel of Fig. S9, we plot the ground state wavefunctions using $N = 12$ basis states. We see that continuum wavefunction matching is working as expected. The ground state $\psi'_0(r)$ agrees with $\psi_0(r)$ beyond some radius R. Also, $\psi'_0(r)$ is approximating $\psi^S(r)$ for $r < R$, up to a constant or proportionality κ that is approximately equal to 1. As expected, the radius R appears to scale with the square root of N. The main difference from the lattice version of wavefunction matching is that there is also an ultraviolet regulator that produces oscillations at small r.

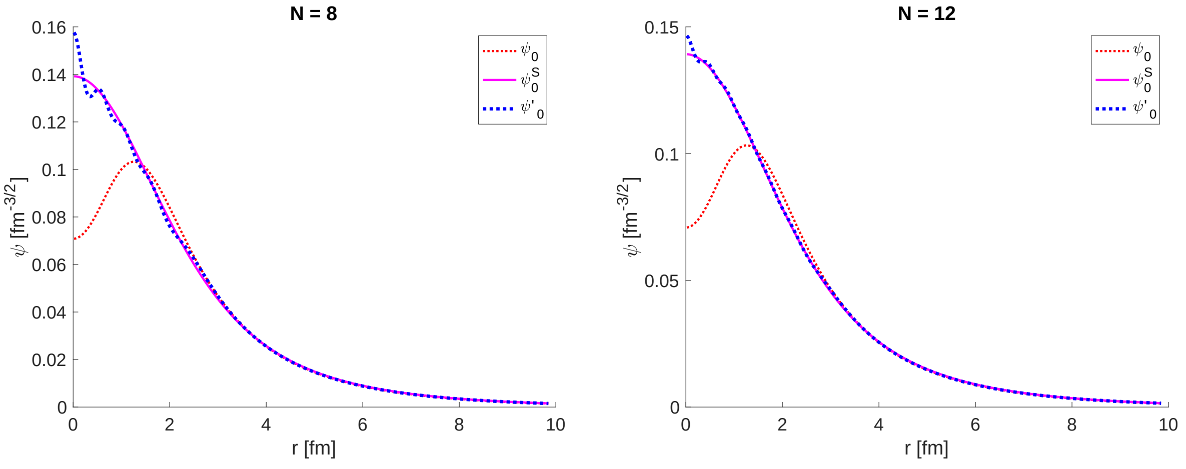

Figure S9. Ground state wavefunctions for continuum wavefunction matching. Left Panel: Ground state wavefunctions for H, H^S, H' using $N = 8$ basis states. **Right Panel:** Ground state wavefunctions for H, H^S, H' using $N = 12$ basis states.

In Table S5, we show wavefunction matching results using Gram-Schmidt orthogonalization for the lowest four S-wave two-body energies with hard wall boundary at $R_{\text{wall}} = 39.46$ fm. From left to right, we present the energies for H (column 1), H' with $N = 4$ (column 2), H' with $N = 4$ using first-order perturbation theory (column 3), H' with $N = 8$ (column 4), H' with $N = 8$ using first-order perturbation theory (column 5), H' with $N = 12$ (column 6), H' with $N = 12$ using first-order perturbation theory (column 7), and H using first-order perturbation theory (column 8). In Table S6, we show wavefunction matching results using Givens rotation for the lowest four S-wave two-body energies with hard wall boundary at $R_{\text{wall}} = 39.46$ fm.

Table S5. Wavefunction matching results using Gram-Schmidt orthogonalization for the lowest four S-wave two-body energies with hard wall boundary at $R_{\rm wall} = 39.46$ fm. From left to right, we present the energies for H (column 1), H' with $N = 4$ (column 2), H' with $N = 4$ using first-order perturbation theory (column 3), H' with $N = 8$ (column 4), H' with $N = 8$ using first-order perturbation theory (column 5), H' with $N = 12$ (column 6), H' with $N = 12$ using first-order perturbation theory (column 7), and H using first-order perturbation theory (column 8). All energies are units of MeV.

E_2^H	$E_2^{H'}$ $N=4$	$E_{2,\rm pert}^{H'}$ $N=4$	$E_2^{H'}$ $N=8$	$E_{2,\rm pert}^{H'}$ $N=8$	$E_2^{H'}$ $N=12$	$E_{2,\rm pert}^{H'}$ $N=12$	$E_{2,\rm pert}^{H}$
-4.5125	-4.5125	-2.9549	-4.5125	-4.1142	-4.5125	-4.4545	-2.6551
0.3344	0.3344	0.3468	0.3344	0.3398	0.3344	0.3392	0.3489
1.3233	1.3233	1.3661	1.3233	1.3441	1.3233	1.3449	1.3728
2.9370	2.9370	3.0175	2.9370	2.9832	2.9370	2.9953	3.0293

Table S6. Wavefunction matching results using Givens rotation for the lowest four S-wave two-body energies with hard wall boundary at $R_{\rm wall} = 39.46$ fm. From left to right, we present the energies for H (column 1), H' with $N = 4$ (column 2), H' with $N = 4$ using first-order perturbation theory (column 3), H' with $N = 8$ (column 4), H' with $N = 8$ using first-order perturbation theory (column 5), H' with $N = 12$ (column 6), H' with $N = 12$ using first-order perturbation theory (column 7), and H using first-order perturbation theory (column 8). All energies are units of MeV.

E_2^H	$E_2^{H'}$ $N=4$	$E_{2,\rm pert}^{H'}$ $N=4$	$E_2^{H'}$ $N=8$	$E_{2,\rm pert}^{H'}$ $N=8$	$E_2^{H'}$ $N=12$	$E_{2,\rm pert}^{H'}$ $N=12$	$E_{2,\rm pert}^{H}$
-4.5125	-4.5125	-2.9549	-4.5125	-4.1142	-4.5125	-4.4545	-2.6551
0.3344	0.3344	0.3464	0.3344	0.3377	0.3344	0.3352	0.3489
1.3233	1.3233	1.3641	1.3233	1.3347	1.3233	1.3263	1.3728
2.9370	2.9370	3.0128	2.9370	2.9595	2.9370	2.9446	3.0293

Since we are applying two-body unitary transformations, the two-body energy spectrum remains the same. $E_2^{H'}$ is exactly the same as E_2^H for each value of N. For both tables, Gram-Schmidt orthogonalization in Table S5 and Givens rotation in Table S6, we see that wavefunction matching accelerates the convergence of perturbation theory for the low-energy states with increasing N. This can be seen by comparing the difference between E_2^H and $E_{2,\rm pert}^H$ and the smaller difference between $E_2^{H'}$ and $E_{2,\rm pert}^{H'}$.

S11 Analysis of Uncertainties due to the Chiral Interactions

We discuss the analysis and quantify the errors which include relevant sources of uncertainty due to the chiral interactions presented in this work. To perform such a complete analysis to estimate the uncertainties for the calculations using chiral interactions, a global parameter search for the LECs of the chiral interactions is required. This task could be extremely difficult as one has to perform a search over a high-dimensional parameter space. Nevertheless, by performing some prior analyses we can reduce both the dimension and the volume of the parameter space to be searched. For instance, the LECs of the two-pion exchange three-body potentials are already fixed from pion–nucleon scattering data, $c_1 = -1.10(3)$, $c_3 = -5.54(6)$ and $c_4 = 4.17(4)$ all in GeV^{-1},[41] and the LECs of two-nucleon potentials can be constrained using the empirical partial wave phase shifts and mixing angles, which help to shrink the parameter space. However, the main difficulty is the determination of the unknown LECs of the three–nucleon forces. As we discuss in the following, we treat these LECs as unknown regression coefficients of an emulator employed with history matching, which has been shown to be an effective approach for parameter searches.[42–45] History matching is an iterative process that identifies and eliminates implausible parts of the input space by measuring implausibility of inputs, shrinks the input space in every iteration, and repeats the non-implausible input search in the smaller input space.

In our calculations we determine the LECs of the 2N chiral interactions at N3LO by fitting the calculated neutron-proton scattering phase shifts and mixing angles on the lattice to the Nijmegen PWA (NPWA).[25] The fundamental sources of uncertainties on these LECs are the systematic errors due to the truncated chiral EFT expansion and the statistical errors provided by the NPWA. In order to estimate the uncertainties, we follow the methodology introduced in Refs.[46,47] The approach involves two components and in the first one, we define the systematic errors of an observable $X(p)$ due to the truncated chiral

EFT expansion at order N^{m}LO and momentum p using the following formula,

$$\Delta X^{\mathrm{N^mLO}}(p) = \max\left(Q^{m+2}|X^{\mathrm{LO}}(p)|, Q^m|X^{\mathrm{LO}}(p) - X^{\mathrm{NLO}}(p)|, \ldots, Q|X^{\mathrm{N^{m-1}LO}}(p) - X^{\mathrm{N^mLO}}(p)|\right), \tag{S49}$$

where Q is the estimated expansion parameter controlling the rate of convergence defined as

$$Q = \max\left(\frac{p}{\Lambda_b}, \frac{M_\pi}{\Lambda_b}\right), \tag{S50}$$

with Λ_b representing the breakdown momentum scale. In the second component, we quantify the statistical errors of the observable $X(p)$ using the estimate

$$\Delta_X = \max\left(\Delta_X^{\mathrm{NPWA}}, |\delta_X^{\mathrm{NijmI}} - \delta_X^{\mathrm{NPWA}}|, |\delta_X^{\mathrm{NijmII}} - \delta_X^{\mathrm{NPWA}}|, |\delta_X^{\mathrm{Reid93}} - \delta_X^{\mathrm{NPWA}}|\right), \tag{S51}$$

where δ_X^i specifies the phase shifts and mixing angles based on different NPWA potentials, and Δ_X^{NPWA} represents the statistical errors of the phase shifts and mixing angles in the NPWA. Our analysis computes the overall errors for the neutron-proton scattering phase shifts and mixing angles as functions of relative momenta. The results for the theoretical error bands are shown in Fig. S10. We show error bands for the cases where there are LECs contributing to that observable at lower orders also. This

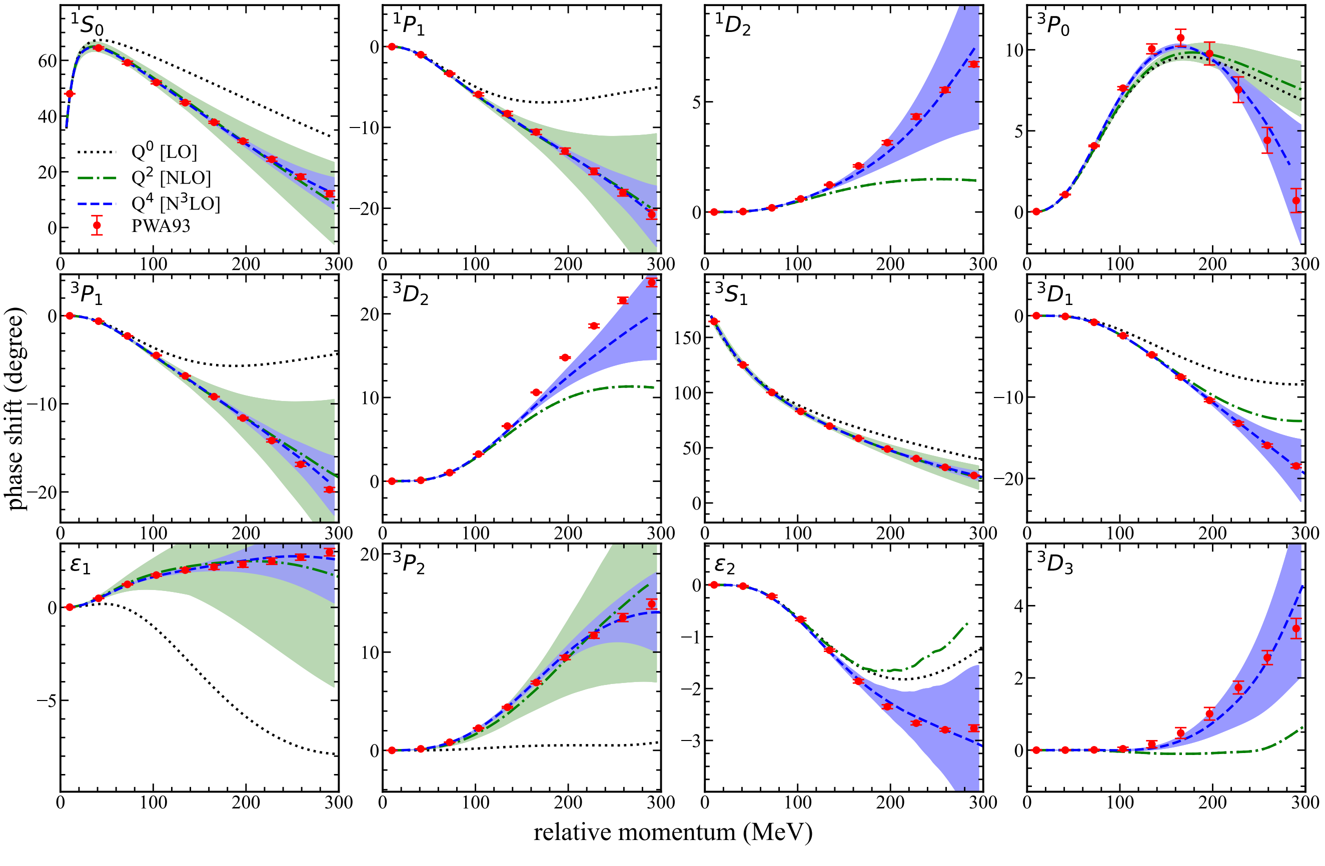

Figure S10. Neutron-proton scattering phase shifts and mixing angles with error bands. The plot of the theoretical error bands for the neutron-proton scattering phase shifts and mixing angles versus the relative momenta.

is done to ensure that the systematic order-by-order convergence is well estimated. Detailed discussions about the convergence of the chiral EFT expansion on the lattice and theoretical errors can be found in Ref.[21]

We use history matching to filter the 2N LECs and 3N LECs that provide an acceptable match between *ab initio* calculations and experimental data for the binding energies of several selected nuclei. The first step in our analysis is to use a Markov Chain Monte Carlo (MCMC) process to obtain a distribution of 2N LECs whose phase shifts and mixing angles corresponds with the N3LO theoretical error bands in Fig. S10. We use the Metropolis algorithm,[48] and our detailed balance function is a Gaussian function of the phase shifts and mixing angles, as prescribed by the mean values and one-sigma deviations for the

N3LO theoretical error bands in Fig. S10. The equilibrium distribution for the 2N LECs obtained from this MCMC process serves as the prior distribution for our history matching analysis. This starting distribution of the 2N LECs is shown as the filled blue bars in Fig. S12, and the correlations are shown in the left panel of Fig. S13.

For notational convenience, we represent the 2N LECs as a vector $\vec{x}$ with components x_j, indexed by j for each 2N LEC. Similarly, we represent the 3N LECs as a vector $\vec{\beta}$ with components β_k, indexed by k for each 3N LEC. We will work with sets of 2N LECs, which we collectively write as $\mathbf{x} = \{\vec{x}^{(i)}\}$. We will consider output observables z_o with index o. We write z_o^{exp} for the experimentally observed values and z_o^{theory} for the theoretical values that we compute using lattice simulations. We define our emulator for observable z_o as

$$f_o(\vec{x}^{(i)}, \vec{\beta}) = z_{o,\text{NP}}^{\text{theory}} + \sum_j x_j^{(i)} \frac{\partial z_o^{\text{theory}}}{\partial x_j^{(i)}} + \sum_k \beta_k \frac{\partial z_o^{\text{theory}}}{\partial \beta_k} \,. \tag{S52}$$

The first term on the right-hand side, $z_{o,\text{NP}}^{\text{theory}}$, corresponds to the non-perturbative contribution to the observable as well as perturbative contributions from operators whose coefficients are not being varied in our history matching analysis. The second term on the right-hand side is the perturbative contribution from the 2N interactions. This term uses 2N inputs from the prior distributions shown in Fig. S12. The third term on the right-hand side is the perturbative contribution from the 3N interactions. For this term we consider the SU(4) symmetric three-nucleon forces $V_{c_E}^{(0)}$, $V_{c_E}^{(1)}$, $V_{c_E}^{(2)}$, $V_{c_E}^{(l)}$, $V_{c_E}^{(t)}$ given in Eqs. (S16), (S18), (S19), and the one-pion-exchange three-nucleon forces $V_{c_D}^{(0)}$, $V_{c_D}^{(1)}$ and $V_{c_D}^{(2)}$ defined in Eq. (S17). In the emulator, Eq. (S52), the derivatives with respect to $x_j^{(i)}$ and β_k stand for the derivatives of observables on the lattice using first-order perturbation theory. Therefore, the emulator uncertainty is the error due to the higher-order perturbations. However, as discussed in Section S13 and shown in Table S8, we are able to reduce this source of systematic error by allowing for variational optimization of the Hamiltonian used to prepare the nuclear many-body wavefunction. We perform this variational optimization so that the remaining systematic error is smaller than the estimated computational error due to stochastic noise, Euclidean time extrapolation, and infinite volume extrapolation.

Let Z_w be the set of outputs that we consider in the wth iteration of history matching, or wave index w. When we emulate our model's behavior for inputs $\mathbf{x} = \{\vec{x}^{(i)}\}$, we use the corresponding experimental observables z_o^{exp} and calibrate the 3N LECs $\vec{\beta}$ so that there is acceptable agreement between our model and the experimental data. For each $\vec{x}^{(i)}$, we define $\vec{\beta}_*(\vec{x}^{(i)})$ to be the optimized $\vec{\beta}$ that achieves the least-squares fit for the relative error,

$$\sum_{o\in Z_w} \left[\frac{f_o(\vec{x}^{(i)}, \vec{\beta}_*(\vec{x}^{(i)})) - z_o^{\text{exp}}}{z_o^{\text{exp}}}\right]^2 = \min_{\vec{\beta}} \sum_{o\in Z_w} \left[\frac{f_o(\vec{x}^{(i)}, \vec{\beta}) - z_o^{\text{exp}}}{z_o^{\text{exp}}}\right]^2 . \tag{S53}$$

It is convenient to rewrite this using vectorized notation,

$$\sum_{o\in Z_w} \left[\frac{f_o(\mathbf{x}, \vec{\beta}_*(\mathbf{x})) - z_o^{\text{exp}}}{z_o^{\text{exp}}}\right]^2 = \min_{\vec{\beta}} \sum_{o\in Z_w} \left[\frac{f_o(\mathbf{x}, \vec{\beta}) - z_o^{\text{exp}}}{z_o^{\text{exp}}}\right]^2 . \tag{S54}$$

When we evaluate Eq. (S54), we impose a constraint on the least squares problem so that only natural sized parameters are allowed for the 3N LECs $\vec{\beta}$. The values are restricted to ensure that the expectation values of individual 3N interactions do not exceed about 30% of the total contribution from the expectation value of the 2N interactions. This constraint is enforced by setting maximum and minimum values for the individual 3N LECs and prevents unphysically large 3N energy cancellations among the different 3N interactions. In our numerical experiments, we observed a significant adverse impact on the results for the excited states in the absence of such a constraint.

Let G_w denote the volume of non-implausible input space, with w indicating the history matching iteration number or wave index. For each output index o, the implausibility measure $I_o(\mathbf{x})$ is a function acting on each element of the set $\mathbf{x} = \{\vec{x}^{(i)}\}$. We use the definition,

$$I_o(\mathbf{x}) = \frac{\left| f_o(\mathbf{x}, \vec{\beta}_*(\mathbf{x})) - z_o^{\text{exp}} \right|}{\sqrt{\text{Var}\left[f_o(\mathbf{x}, \vec{\beta}_*(\mathbf{x}))\right] + \text{Var}\left[\varepsilon_o(\mathbf{x})\right]}} \,. \tag{S55}$$

Here, $\text{Var}\left[f_o(\mathbf{x}, \vec{\beta}_*(\mathbf{x}))\right]$ is the variance of $f_o(\mathbf{x}, \vec{\beta}_*(\mathbf{x}))$ over the set of elements $\{\vec{x}^{(i)}\}$, and $\text{Var}[\varepsilon_o(\mathbf{x})]$ corresponds to estimates of the mean squared error due to Monte Carlo stochastic noise, Euclidean time extrapolation, and infinite volume extrapolation.

Any particular element $\vec{x}^{(i)}$ of the set $\mathbf{x}$ that gives a large value for I_o is considered implausible. This means that this input is unlikely to produce an acceptable match between the calculated outputs and experimental data. For this purpose, we define the maximum implausibility measure $I_M(\mathbf{x})$ as

$$I_M(\mathbf{x}) = \max_{o \in Z_w} I_o(\mathbf{x}), \tag{S56}$$

where Z_w is again the set of outputs that we consider in the wth iteration, or wave index w. We discard the input $\vec{x}^{(i)}$ if $I_M(\vec{x}^{(i)}) > c$, where c is our cutoff threshold for non-implausibility. Following Pukelsheim's 3–sigma rule,[49] we use $c = 3$. This corresponds to 95% of the probability distribution lying within $\pm 3\sigma$ of the mean value for a random probability distribution with variance σ. We refer the reader to Ref.[44] for a general description of history matching and Ref.[45] for a nuclear physics application.

In the following, we discuss the application of history matching to our problem. We perform the history matching analysis for the 2N LECs in separate four waves. With each successive wave, we include two additional 3N interactions, with parameters tuned to produce the best fit of the binding energies for the selected nuclei. For WAVE 1, we use $V_{cE}^{(0)}$ and $V_{cD}^{(0)}$. For WAVE 2, we add $V_{cE}^{(l)}$ and $V_{cE}^{(t)}$. $V_{cE}^{(1)}$ and $V_{cD}^{(1)}$ are added in WAVE 3, and $V_{cE}^{(2)}$ and $V_{cD}^{(2)}$ are added in WAVE 4. The chosen ordering is consistent with the ordering of descending importance found in Section S18. However, we have chosen to include $V_{cE}^{(1)}$ and $V_{cD}^{(1)}$ together in the same wave rather than $V_{cE}^{(1)}$ and $V_{cE}^{(2)}$.

WAVE 1 In the first wave, the implausibility measure is run over outputs $Z_1 = \{^3\text{H}, ^4\text{He}\}$. For the first wave, we also restrict the inputs to those 2N LECs associated with the $^1S_0$ and $^3S_1 - ^3D_1$ channels. This choice is based on the fact that the S-wave interactions are dominant for the nuclei ^{3}H and ^{4}He. The other waves will have no such restrictions on the inputs. The non-implausible inputs $\mathbf{x}$ are selected from the prior distributions for the 2N LECs shown by blue bars in Fig. S12. We refer to this set of non-implausible inputs as the initial volume G_0. We calculate the model outputs using Eq. (S52) and Eq. (S54) using the 3N interactions $V_{cE}^{(0)}$ and $V_{cD}^{(0)}$ only. The implausibility measures $I_M(\mathbf{x})$ are calculated using the maximum implausibility over outputs, and the implausibility cutoffs are imposed to remove implausible data. The remaining elements of G_0 define the new non-implausible volume, which we label as G_1. The results of the fitted (predicted) observables are shown with blue open (filled) circles in Fig. S11. We plot the relative deviations between the model outputs and experimental data written as a percentage. From Fig. S11, we see that the deviations for all the predicted outputs are negative, corresponding with underbinding. This provides information for choosing the outputs to be included in the next wave.

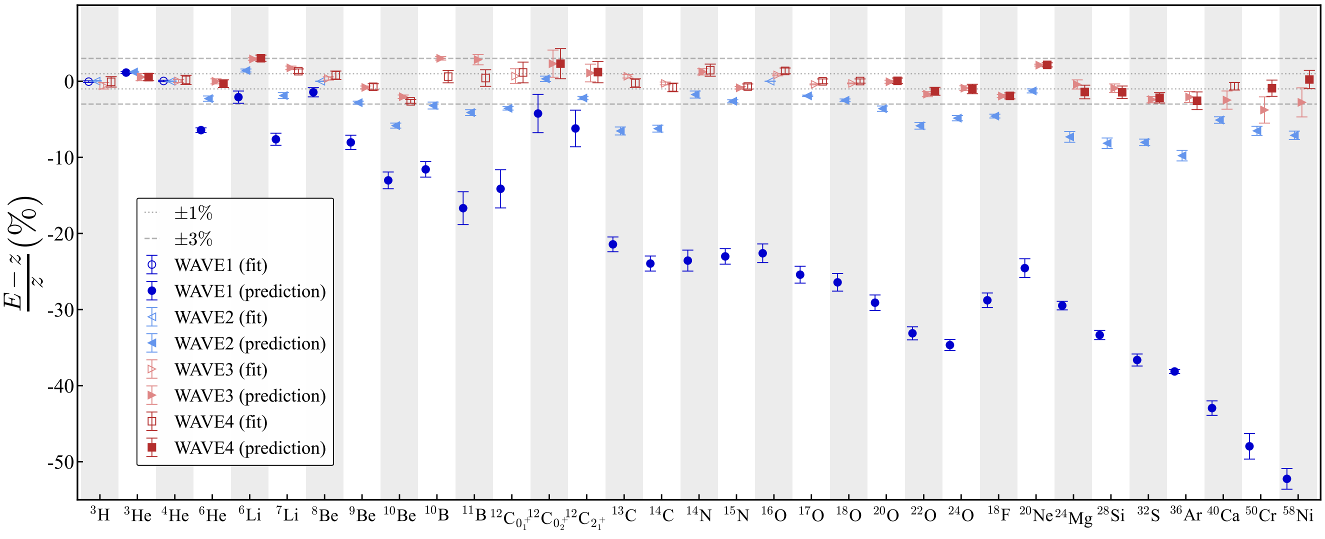

Figure S11. Relative deviations for binding energies. Plot of relative deviations between the model outputs and experimental data per nucleon for history matching iterations from WAVE 1 to WAVE 4.

WAVE 2 At this wave, we run the implausibility measure over outputs $Z_2 = Z_1 \bigcup \{^8\text{Be}, ^{16}\text{O}\}$. This choice is motivated by the strong evidence that these additional nuclei have alpha cluster substructures in various geometric shapes. We use all of the 2N LECs as inputs. The non-implausible parameter space for the $^1S_0$ and $^3S_1 - ^3D_1$ channels are given by the volume

G_1. For all of the other channels, the non-implausible data are selected from the corresponding prior distributions shown in Fig. S12. For this wave, we use two additional 3N interactions $V_{c_E}^{(l)}$ and $V_{c_E}^{(t)}$ to perform the least squares regression in Eq. (S54). The implausibility cutoffs are imposed to remove implausible data, and the remaining set of non-implausible data is called G_2. The results of the fitted (predicted) observables at WAVE 2 are shown with light-blue open (filled) left-pointing triangles in Fig. S11. We find that using four 3N interactions in WAVE 2 already gives a decent description for light nuclei with $N = Z$, while the deviations for other nuclei are significant.

WAVE 3 For this wave, we run the implausibility measure over outputs $Z_3 = Z_2 \bigcup \{^{12}\text{C}, ^{13}\text{C}, ^{14}\text{C}, ^{17}\text{O}, ^{18}\text{O}\}$. The non-implausible input volume is set by G_2. We perform the least squares fits in Eq. (S54) using two additional 3N interactions, $V_{c_E}^{(1)}$ and $V_{c_D}^{(1)}$. The resulting non-implausible volume as denoted as G_3. We show the results of the fitted (predicted) observables at WAVE 3 in Fig. S11 with pink open (filled) right-pointing triangles. The results in Fig. S11 show that the agreement between the calculated and experimental data is fairly good for most of the nuclei.

WAVE 4 For this wave, we run the implausibility measure over outputs $Z_4 = Z_3 \bigcup \{^{7}\text{Li}, ^{9}\text{Be}, ^{10}\text{Be}, ^{10}\text{B}, ^{11}\text{B}, ^{14}\text{N}, ^{15}\text{N}, ^{40}\text{Ca}\}$ and include the last two additional 3N interactions, $V_{c_E}^{(2)}$ and $V_{c_D}^{(2)}$. The final non-implausible volume is denoted as G_4. The results of the fitted (predicted) observables are shown with red open (filled) squares in Fig. S11. We see that the relative deviations are less than or equal to 3% percent for all of the nuclei.

The 2N LECs corresponding to the non-implausible volume G_4 are displayed as hollow red bars in Fig. S12. The filled blue bars in Fig. S12 are the prior distributions obtained from MCMC sampling of the 2N LECs according to the N3LO theoretical error bands in Fig. S10, and the dashed lines give the best fit values for the 2N LECs. The corresponding correlations are shown in Fig. S13. In Table S7, we give the binding energies with uncertainties for each of the nuclei and for each of the four waves. The central values and uncertainty estimates correspond to the mean values and standard deviations of the binding energies associated with points in the non-implausible volume.

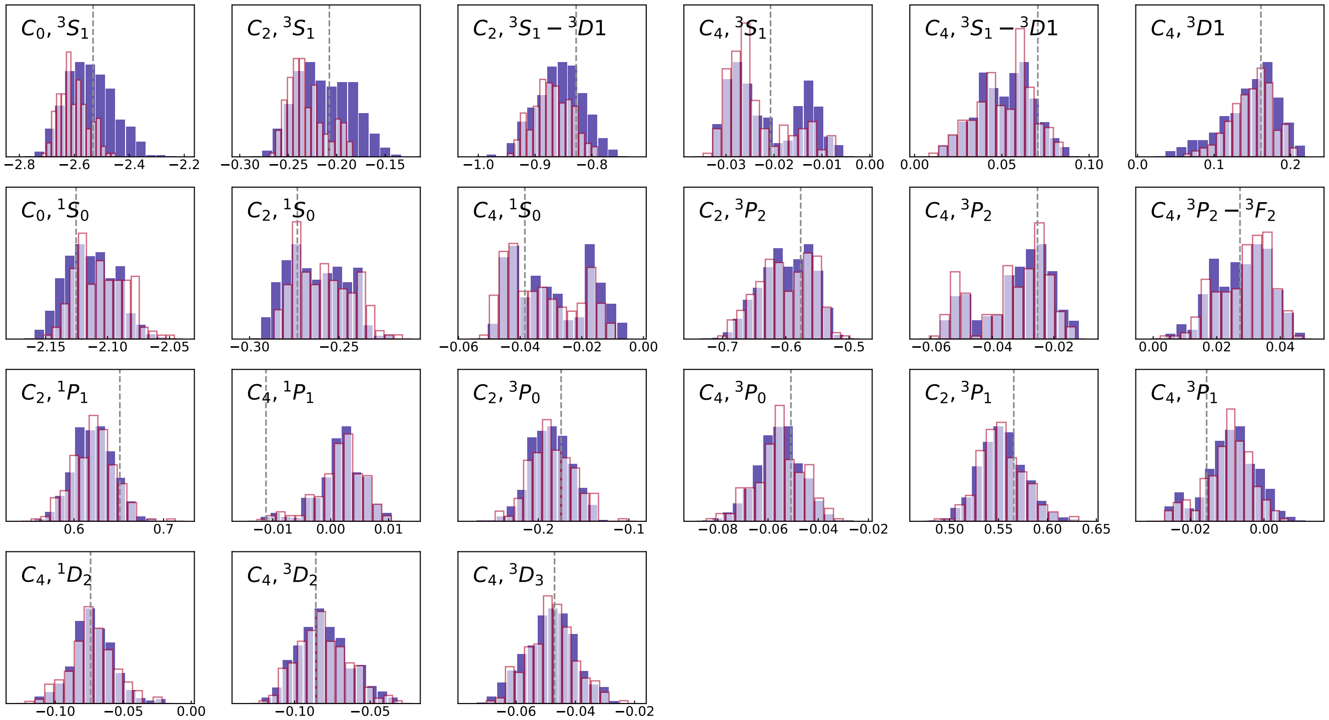

Figure S12. Prior distributions and final non-implausible volumes for the 2N LECs. The filled blue bars are prior distributions obtained from MCMC sampling of the 2N LECs according to the N3LO theoretical error bands in Fig. S10. The hollow red bars are the non-implausible volumes for the 2N LECs obtained after four waves of the history matching analysis. The horizontal axes are in lattice units.

In Fig. S14 we plot the distributions for the 3N coefficients corresponding to the non-implausible volume after WAVE 4. The horizontal axis is in lattice units. The dashed lines are 3N coefficient values that produce the best fit for the fitted binding energies shown in the main text, when using 2N LECs set to the best fit values for the scattering phase shifts and mixing

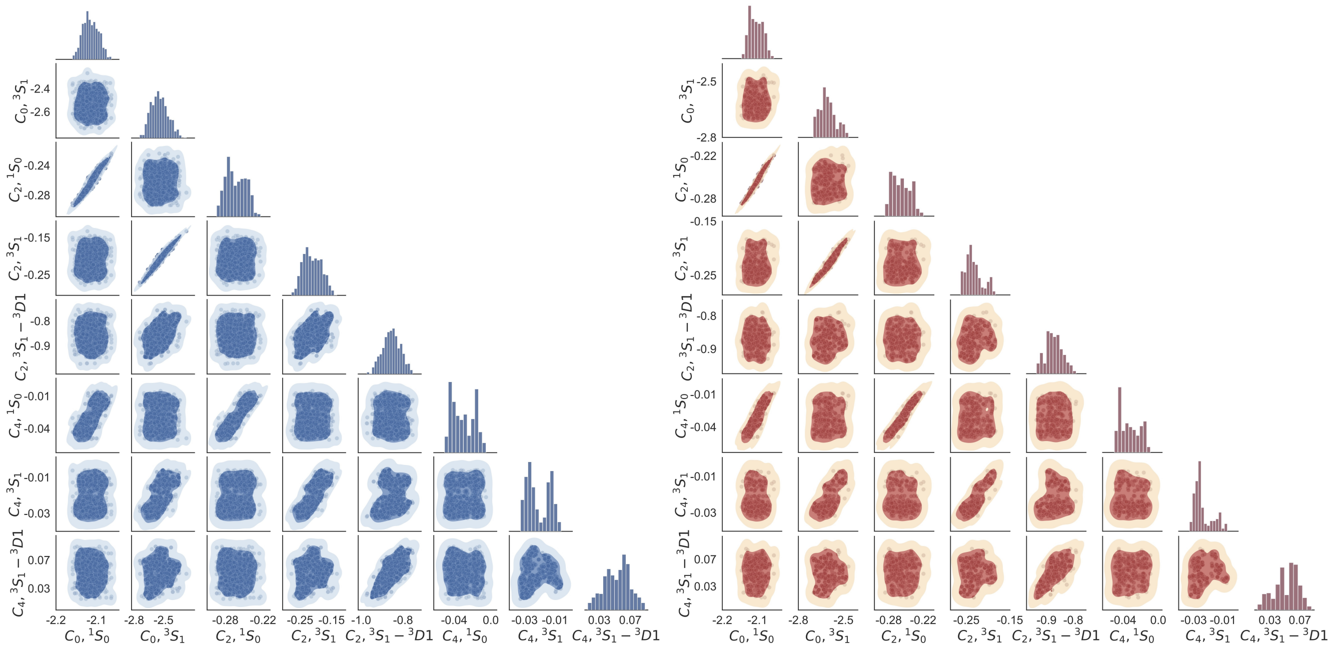

Figure S13. Prior and final non-implausible volume correlations for the 2N LECs. The left panel shows correlations in the prior distributions obtained from MCMC sampling of the 2N LECs according to the N3LO theoretical error bands in Fig. S10. The right panel shows correlations in the non-implausible volumes for the 2N LECs obtained after four waves of the history matching analysis. The horizontal and vertical axes are in lattice units.

angles. In some cases, we can see the importance of the maximum and minimum boundary constraints on the 3N interaction coefficients. See the discussion about constraints on the 3N interaction coefficients directly after Eq. (S54).

S12 *Ab Initio* Chiral Effective Field Theory

Over the past two decades, nuclear *ab initio* calculations have progressed greatly. The widespread adoption of nuclear forces from chiral effective field theory[50–57] laid the foundation for these developments. More specifically, the inclusion of chiral two-nucleon forces to higher orders[53–55,58] and the three-nucleon[52,59–61] forces have shown to be key elements for high-quality calculations. The strong short-range repulsion in nuclear potentials is often softened by a renormalization procedure.[62] Besides Nuclear Lattice Effective Field Theory (NLEFT), there are many other promising *ab initio* approaches being used to calculate the properties of few- and many- nucleon systems. Full configuration methods like no-core shell model (NCSM),[63–66] symmetry-adapted NCSM[67,68] and quantum Monte Carlo in several different varieties[69–73] were able to extend their reach into the lower *sd*-shell.

With controlled truncations, a variety of computationally efficient techniques were developed, like self-consistent Green's functions,[74] closed-shell many-body perturbation theory,[75,76] coupled cluster,[77] in-medium similarity renormalization group (IMSRG),[78] and *ab initio* no-core Monte Carlo shell model.[79,80] In the meantime, effective shell model Hamiltonians can be constructed by open-shell MBPT,[81–83] valence-space IMSRG[84,85] and shell model coupled cluster.[86,87] To include continuum effects in weakly bound nuclei, several methods were also well established, like the no-core shell model with continuum (NCSMC),[88] complex CC,[89] no-core Gamow shell model (GSM),[90,91] Gamow IMSRG[92] and GSM with realistic forces.[93,94] It should be noted that the level of the approximation is not the same in all these different approaches, but a detailed comparison goes beyond the scope of this paper.

S13 Many-Body Convergence of *Ab Initio* Chiral Effective Field Theory

As noted in the previous section, *ab initio* calculations of the nuclear many-body problem using chiral effective field theory have made great strides over the past two decades. However, the ultimate goal of *ab initio* nuclear structure theory is to predict the low-energy properties of all atomic nuclei with reliable and systematically improvable errors that are small enough to be useful for experiments. Despite the impressive recent advances, we are still far away from reaching this level of reliability and predictive value.

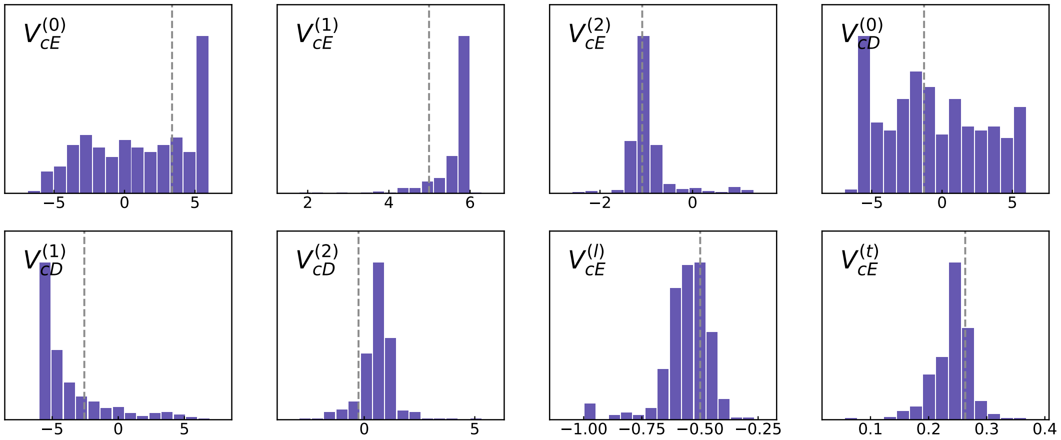

Figure S14. Distributions for the 3N coefficients corresponding to the non-implausible volume after WAVE 4. The dashed lines are the best fit 3N coefficients corresponding to 2N LECs giving the best fit for the scattering phase shifts and mixing angles. The horizontal axis is in lattice units.

Some recent discussions on the current status of *ab initio* nuclear theory are presented in Ref.[95,96] The most well-known roadblock currently facing *ab initio* calculations is the difficulty of maintaining high-fidelity two-nucleon phase shifts and mixing angles and describing the saturation energy and density of symmetric nuclear matter as well as the binding energies and charge radii of light and medium-mass nuclei. Previous *ab initio* nuclear structure calculations have either not addressed some of the relevant observables or require further improvement in one or more of these areas.

A fundamental problem in applying chiral effective field theory to the nuclear many-body problem is that the detailed features of the short-distance regulator often has an impact on the final results. Regardless of the computational method or choice of chiral interaction, many practitioners in *ab initio* nuclear structure theory have independently found that the properties of medium-mass nuclei and nuclear matter depend on the choice of regulator used for the three-nucleon interactions. Why this happens and how it can be systematically fixed is one of the main results of this work.

The results in Ref.[33,34] showed that the range and locality of the nuclear interactions have a strong influence on nuclear binding. The $\alpha\alpha$ interaction is highly sensitive to the range and locality of the nucleonic interactions. These same arguments apply to other interactions involving α particles and nucleons (N). Several groups have observed that the αN scattering phase shifts in the $^{3/2}$P and $^{1/2}$P channels are sensitive to the three-nucleon interactions.[97,98] NLEFT calculations performed for this study have confirmed this result and have also found significant sensitivity to the locality of the three-nucleon interactions.

The strong correlation between accurate nuclear binding energies and the interactions among α particles and nucleons can be seen empirically from the analysis of the three-nucleon interactions in Section S18. The analysis is postponed until the last section due to the lengthy tables of fit results. In Table S13, we see that using only the simplest three-body interactions $V^{(0)}_{c_E}$ and $V^{(0)}_{c_D}$ does a poor job in reproducing the nuclear binding energies. The fundamental problem can be diagnosed quite easily as arising from the fact that energy differences such as $E^6_{12,0^+_1} - 3E^2_4$, $E^8_{16} - 4E^2_4$, $E^2_6 - E^2_4$, $E^4_9 - E^4_8$, $E^6_{13} - E^6_{12}$, and $E^6_{14} - E^6_{12}$ are significantly higher than they should be. Given the known cluster structure for many of these light nuclei, we conclude that the interactions among the α particles and between α particles and nucleons need to be more attractive. We note that the small energy differences between the ^{8}Be and ^{5}He ground state resonances and their nearby continuum thresholds are comparable to the uncertainties of the lattice calculations. Bound light nuclei with cluster substructures are therefore more convenient when computing energy differences.

Our analysis can be put into the framework of cluster effective field theory (cluster EFT) for α particles and nucleons.[99–102] The two-cluster interactions are $\alpha\alpha$ and αN, and the three-cluster interactions are αNN, $\alpha\alpha\alpha$, and $\alpha\alpha N$. The αNN combination has two possible isospin channels. The six interactions are illustrated in Fig. S15 in panels a, b, c, d, e, and f respectively. While cluster EFT is not designed to describe the structure of heavy nuclei or nuclear matter at saturation, it provides a useful diagnostic tool for understanding how regulator-dependent errors grow as we increase the number of nucleons from the smallest few-body systems to light and medium-mass nuclei. Given that there are six independent cluster EFT interactions, we expect that no more than six independent parameters must be tuned to remove significant regulator-dependent artifacts from the *ab initio* nuclear structure calculations.

Given the importance of the $\alpha\alpha$ interaction for the binding of nuclei with equal numbers of protons and neutrons, we expect that the first parameter that must be tuned is the $\alpha\alpha$ interaction. This statement was already demonstrated in Ref.[17] In that

Table S7. Nuclear binding energies with history matching uncertainties. Nuclear binding energies of selected nuclei with uncertainties using history matching for WAVE 1 through WAVE 4. The nuclear binding energies used in the fit for each wave are indicated with bold font.

Nuclei	WAVE 1	WAVE 2	WAVE 3	WAVE 4	Experiment (MeV)
^{3}H	**8.48(1)**	**8.48(1)**	**8.43(4)**	**8.47(6)**	8.48
^{3}He	7.81(1)	7.81(1)	7.76(4)	7.76(4)	7.72
^{4}He	**28.31(2)**	**28.30(1)**	**28.31(5)**	**28.35(17)**	28.30
^{6}He	27.39(9)	28.60(10)	29.27(10)	29.18(15)	29.27
^{6}Li	31.32(26)	32.44(6)	32.93(5)	32.96(13)	31.99
^{7}Li	36.25(31)	38.50(15)	39.93(9)	**39.76(11)**	39.24
^{8}Be	55.68(34)	**56.50(1)**	**56.71(12)**	**56.94(31)**	56.50
^{9}Be	53.50(54)	56.53(11)	57.70(11)	**57.75(24)**	58.17
^{10}Be	56.51(71)	61.18(21)	63.65(15)	**63.25(16)**	64.97
^{10}B	57.25(66)	62.68(29)	66.70(15)	**65.15(53)**	64.75
^{11}B	63.49(165)	73.06(29)	78.37(52)	**76.52(83)**	76.20
$^{12}\text{C}_{0_1^+}$	79.13 (231)	88.88 (21)	**92.79 (89)**	**93.23 (125)**	92.16
$^{12}\text{C}_{0_2^+}$	80.93(213)	84.78(30)	86.46(151)	86.47(167)	84.51
$^{12}\text{C}_{2_1^+}$	82.28(211)	85.79(19)	88.67(102)	88.78(123)	87.72
^{13}C	76.30(94)	90.77(51)	**97.74(22)**	**96.88(55)**	97.11
^{14}C	80.06(104)	98.72(48)	**104.98(19)**	**104.43(59)**	105.28
^{14}N	79.99(144)	102.83(49)	105.96(43)	**106.19(83)**	104.66
^{15}N	88.91(118)	112.46(23)	114.49(20)	**114.69(39)**	115.49
^{16}O	98.77 (157)	**127.62(1)**	**128.69(15)**	**129.36(48)**	127.62
^{17}O	98.27(146)	129.23(13)	**131.22(9)**	**131.75(41)**	131.76
^{18}O	102.87(162)	136.31(25)	**139.42(8)**	**139.85(39)**	139.81
^{20}O	107.32(154)	145.92(53)	151.27(30)	151.45(56)	151.37
^{22}O	108.36(139)	152.54(72)	159.29(54)	159.89(85)	162.03
^{24}O	110.01(121)	160.22(58)	166.85(53)	166.67(104)	168.38
^{18}F	97.83(132)	131.08(34)	134.70(29)	134.73(54)	137.37
^{20}Ne	121.20(198)	158.61(42)	164.05(20)	164.11(44)	160.65
^{24}Mg	139.81 (111)	183.76(141)	197.43(120)	195.46(177)	198.26
^{28}Si	157.66(144)	217.27(163)	234.38(133)	233.11(195)	236.54
^{32}S	172.22(215)	249.96(116)	265.22(112)	265.87(185)	271.78
^{36}Ar	189.75(83)	276.74(212)	300.37(221)	298.86(357)	306.72
^{40}Ca	195.13(326)	324.63(147)	333.59(410)	**339.82(170)**	342.05
^{50}Cr	226.35(733)	406.69(260)	418.63(752)	431.07(464)	435.05
^{58}Ni	241.84(691)	470.47(279)	492.41(968)	507.62(608)	506.46

work, three of the interaction parameters were tuned according to the properties of few-body systems with up to three nucleons. By tuning only one additional parameter associated with the strength of the local smearing regulator, the properties of light nuclei, medium-mass nuclei, and neutron matter were reproduced with no more than a few percent error. The success of this simple approach provides evidence that one parameter, the strength of the $\alpha\alpha$ interaction, plays a dominant role in the binding of nuclei with equal numbers of protons and neutrons.

We can see the same underlying physics in our analysis here. Comparing Table S13 and Table S14, we see that adding just one additional three-nucleon interaction with local smearing increases the binding of nuclei with even and equal numbers of protons and neutrons. With the exception of the last row in Table S14 corresponding to the poorest fit, we see that the fits using $V_{c_D}^{(2)}$, $V_{c_E}^{(1)}$, $V_{c_E}^{(2)}$, $V_{c_E}^{(l)}$, and $V_{c_E}^{(t)}$ all have similar features. This can be seen in the lowering of the energy differences $E^6_{12,0_1^+} - 3E^2_4$ and $E^8_{16} - 4E^2_4$, which are strongly correlated with the $\alpha\alpha$ interaction. In each case, the energy differences $E^6_{12,0_1^+} - 3E^2_4$ and $E^8_{16} - 4E^2_4$ are brought much closer to their physical values, and the root-mean-square error for the binding energy per nucleon drops from 1.2 MeV to about 0.4 MeV.

As we include more three-nucleon interactions in Table S15, Table S16, Table S17, Table S18, and Table S19, the descriptions of the energies differences get better. The best root-mean-square error for the binding energy per nucleon drops to 0.29 MeV, 0.13 MeV, 0.11 MeV, 0.10 MeV, and 0.08 MeV respectively. We see that, after including three additional three-nucleon interactions, we reach a plateau of about 0.1 MeV error in the binding energy per nucleon. From this analysis, we conclude there are three or four independent parameters that must be tuned in order to remove significant regulator-dependent artifacts from the *ab initio* calculations. This conclusion can also be drawn from the history matching analysis. In Fig. S11, we see that there is only minor improvement in the size of the estimated errors and the accuracy of the predictions when going

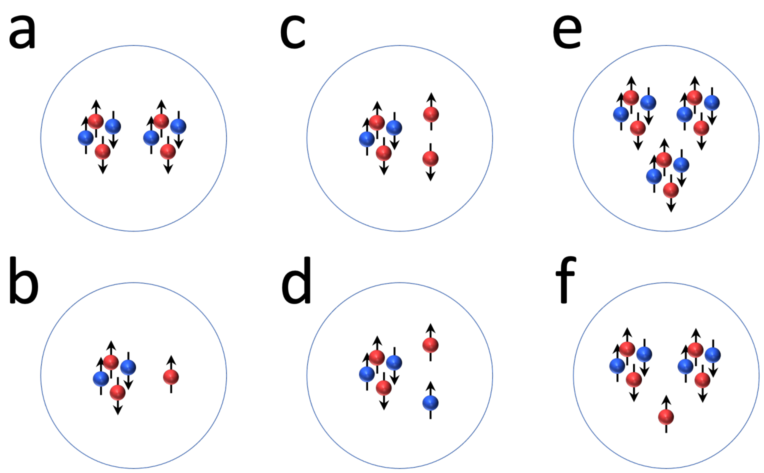

Figure S15. Cluster effective field theory interactions. Panel a: $\alpha\alpha$. **Panel b:** αN. **Panel c:** αNN with isospin 1. **Panel d:** αNN with isospin 0. **Panel e:** $\alpha\alpha\alpha$. **Panel f:** $\alpha\alpha N$.

beyond WAVE 3.

The error plateau of 0.1 MeV energy per nucleon indicates that there are some additional sources of error at this level. For most calculations, the Monte Carlo error and Euclidean time extrapolation is about a factor of two smaller than this amount. The 0.1 MeV energy per nucleon is more consistent with the size of the errors from higher corrections in perturbation theory. We can estimate this error by varying the interaction coupling for the Hamiltonian used to prepare the nuclear many-body wavefunction and noting the variation in the expectation value of the N3LO Hamiltonian H' with three-nucleon interactions included. We note that first-order perturbation theory for the energy is equivalent to a variational calculation for the energy. In Table S8, we present results for several example nuclei at $L_t = 741$ lattice time steps, or Euclidean time duration 0.741 MeV^{-1}. From the variations in the expectation value of H', we see that an error estimate of about 0.1 MeV per nucleon is a reasonable estimate of the size of the higher-order perturbation theory corrections to the energy. At the same time, we can also reduce this systematic error significantly by variational optimization of the Hamiltonian used to prepare the nuclear many-body wavefunction.

Table S8. Variational dependence of binding energies on coupling used to prepare trial state. We show the variation of the expectation value of N3LO Hamiltonian H' with three-nucleon interactions as a function of the interaction coupling $c_{\mathrm{SU}(4)}$ for the Hamiltonian used to prepare the nuclear many-body wavefunction. Results are shown for several example nuclei.

Nucleus	$c_{\mathrm{SU}(4)}$ [10^{-6} MeV^{-2}]	$\langle H' \rangle$ [MeV]
^{3}H	-0.36	-8.40(8)
^{3}H	-0.39	-8.43(9)
^{3}H	-0.42	-8.29(11)
^{4}He	-0.36	-28.30(7)
^{4}He	-0.39	-28.32(7)
^{4}He	-0.42	-28.23(8)
^{14}C	-0.39	-101.1(2)
^{14}C	-0.42	-101.5(4)
^{14}C	-0.48	-100.8(4)
^{17}O	-0.39	-130.3(7)
^{17}O	-0.42	-131.0(9)
^{17}O	-0.48	-129.6(14)
^{32}S	-0.36	-259(1)
^{32}S	-0.39	-263(1)
^{32}S	-0.42	-261(1)

S14 Properties of the Three-Nucleon Interactions

The three-nucleon interactions with regulator-dependent coefficients are illustrated in Fig. S16. Each of these interactions corresponds to the usual c_D and c_E three-nucleon terms in chiral effective field theory, but with different choices for the local smearing regulator. In essence, we are engineering the regulator structure of the c_D and c_E terms to cancel the dominant regulator-dependent errors that arise in *ab initio* nuclear structure calculations.

In Fig. S16, $V_{c_D}^{(0)}$ shown in panel a is the c_D term with no additional local smearing. $V_{c_D}^{(1)}$ in panel b is the c_D term with local smearing up to one lattice spacing. $V_{c_D}^{(2)}$ in panel c is the c_D term with local smearing up to $\sqrt{2}$ times the lattice spacing. $V_{c_E}^{(0)}$ in panel d is the c_E term with no additional local smearing. $V_{c_E}^{(1)}$ in panel e is the c_E term with local smearing up to one lattice spacing. $V_{c_E}^{(2)}$ in panel f is the c_E term with local smearing up to $\sqrt{2}$ times the lattice spacing. $V_{c_E}^{(l)}$ in panel g is the c_E term with local smearing where the three nucleons are at different sites with a prolate configuration. This corresponds to three nucleons lying along a line with each nucleon one lattice unit apart from their neighbor(s). $V_{c_E}^{(t)}$ in panel h is the c_E term with local smearing where the three nucleons are on different sites with an oblate configuration. This corresponds to three nucleons forming an equilateral triangle with side length equal to $\sqrt{2}$ lattice units.

Although the three nucleons sit on different lattice sites, the three-nucleon configurations for $V_{c_E}^{(l)}$ and $V_{c_E}^{(t)}$ are still very compact. The root-mean-square radius for the $V_{c_E}^{(l)}$ configuration is 1.07 fm, and the root-mean-square radius for the $V_{c_E}^{(t)}$ configuration is also 1.07 fm. In Ref.[11] it was shown that all the low-lying states of ^{12}C could be described as either an equilateral triangle of alpha clusters or an obtuse triangle of alpha clusters. The oblate shape for $V_{c_E}^{(t)}$ and the prolate shape for $V_{c_E}^{(l)}$ are designed to have some overlap with α particles in such arrangements.

In the future, it may be possible to use NLEFT calculations to tune the three-nucleon coefficients to reproduce the physical scattering properties of α clusters and nucleons using the adiabatic projection method.[28] However, the approach we employ here with binding energies of selected nuclei has the advantage that it is simple and can be readily used with other *ab initio* methods.

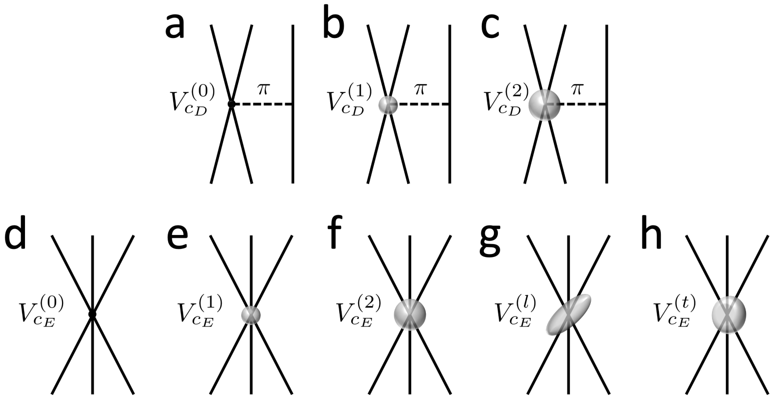

Figure S16. Three-nucleon interactions with regulator-dependent coefficients. Panel a: $V_{c_D}^{(0)}$. Panel b: $V_{c_D}^{(1)}$. Panel c: $V_{c_D}^{(2)}$. Panel d: $V_{c_E}^{(0)}$. Panel e: $V_{c_E}^{(1)}$. Panel f: $V_{c_E}^{(2)}$. Panel g: $V_{c_E}^{(l)}$. Panel h: $V_{c_E}^{(t)}$.

S15 New Chiral Effective Field Theory Interactions with Wavefunction Matching

We have discussed many computational aspects of wavefunction matching and its ability to accelerate the convergence of perturbation theory in *ab initio* many-body calculations. In this section, we discuss the use of wavefunction matching as a theoretical tool for exploring new chiral effective field theory interactions with different short-distance regulator properties. By changing the high-fidelity Hamiltonian H, the simple Hamiltonian H^S, the wavefunction matching radius R, and/or the choice of unitary transformation U (Gram-Schmidt orthogonalization, Givens rotation, etc.), we can construct a large class of transformed Hamiltonians H'. As an important bonus, the accelerated convergence of perturbation theory provides an efficient method for performing the calculations. This allows for the exploration of a very large class of high-fidelity chiral interactions with a wide range of different short-distance regulators.

In Fig. S2, we showed that the ^{3}H and ^{4}He binding energies changed very little when varying R from 2.94 fm to 3.72 fm. We argued in Section S6 that when R exceeds r_Δ, the dependence on R only appears in high-momentum modes. While there

is no noticeable effect on the low-energy physics of few-nucleon systems, these high-momentum modes can impact nuclear many-body properties. As we have argued in Section S13, this may happen if the $\alpha\alpha$ interaction and/or other cluster interactions are impacted by the change in R. This is in fact what happens.

As R is varied from 2.94 fm to 3.72 fm, the binding energy of ^{12}C decreases by about 6 MeV while the binding energy ^{16}O decreases by about 12 MeV. This is the physics of the quantum phase transition studied in Ref.[8] By changing the $\alpha\alpha$ interaction, one can produce a quantum phase transition from a nuclear liquid to a Bose gas of α particles. In that study, the two-nucleon phase shifts and few-nucleon properties were kept approximately the same. Here, the two-nucleon phase shifts remain exactly the same, while the binding energies for ^{3}H and ^{4}He change by no more than 1%. Wavefunction matching provides a new theoretical tool for exploring new chiral interactions with different short-distance regulators. Similar to what we have done in this work, we expect that the dependence on R in nuclear many-body systems can be removed by tuning the regulator structure of the three-nucleon interactions. This work is currently in progress. It is part of a larger program to show that one can produce different high-fidelity chiral interactions with different regulator structures using wavefunction matching and then cancel regulator-dependent errors by tuning the regulator structure of the three-nucleon interactions.

From the nuclear matter results shown in the main text, we see that the energy per nucleon with two-nucleon interactions only does not go below -10 MeV. This can be interpreted as the $\alpha\alpha$ interaction not having enough attraction. The addition of almost any locally smeared three-nucleon interaction fixes the problem quite easily. The size of this correction is about 6 MeV per nucleon at the saturation density. Given that the three-nucleon interactions appear at NNLO or $O(Q^3)$ and the Fermi momentum of about 260 MeV, this contribution is of natural size if the expansion parameter is Q/Λ with $\Lambda \sim \pi/a = 471$ MeV.

For light nuclei, the relevant momentum scale Q is approximately the pion mass. The natural size for the contribution of the three-nucleon interactions is about 1 MeV per nucleon. This is indeed what is observed in the lattice calculations. For medium-mass nuclei, the three-nucleon contribution to the binding energy is intermediate between the 1 MeV per nucleon for light nuclei and 6 MeV per nucleon at the saturation density.

S16 Charge Radii

In our calculations of the root-mean-square charge radii, we use the relation,

$$R_{\rm ch}^2 = \langle r_{pp}^2 \rangle + R_p^2 + \frac{N}{Z} R_n^2 + \frac{3}{4m_p^2}, \tag{S57}$$

where $\langle r_{pp}^2 \rangle$ is the mean-square point proton radius, R_p^2 is 0.7056 fm^2,[103,104] R_n^2 is -0.105 fm^2,[105] and m_p is 938.27 MeV. We have not included relativistic spin-orbit corrections or two-nucleon current contributions. As the size of the computational errors for the lattice calculations are reduced in the future, these smaller contributions will also be included.

In Table S9 we show the computed charge radii of selected nuclei using history matching for WAVE 1 through WAVE 4. The central values and uncertainty estimates correspond to the mean values and standard deviations of the radii associated with points in the non-implausible volume. For comparison, we also show the empirical values. We note that the history matching analysis does not use any charge radii in determining the implausibility measures. Nevertheless, the overall description of the charge radii in Table S9 are improving with each successive wave as more features of the nuclear interaction are determined. When compared with the experimental values, the RMS deviations for the charge radii in Table S9 are 0.117(19) fm for WAVE 1, 0.097(10) fm for WAVE 2, 0.066(16) fm for WAVE 3, and 0.057(14) fm for WAVE 4. These estimates take into account the uncertainties of the history matching results in Table S9. If we use only the central values for the charge radii in Table S9, the corresponding RMS deviations are 0.113 fm for WAVE 1, 0.093 fm for WAVE 2, 0.053 fm for WAVE 3, and 0.033 fm for WAVE 4. We see that as more data for the nucleon-nucleon interaction and binding energies are included, the predictions of the charge radii systematically improve. This is exactly what we would like to see happen in *ab initio* calculations.

S17 Details of Results

In this section, we give numerical details of the results presented in the main text. We have calculated results for the ground state and excited state energies as well as the charge radii of some selected nuclei up to $A = 58$, pure neutron matter, and symmetric nuclear matter at N3LO in chiral effective field theory using wavefunction matching. In Table S10 we present the numerical details of the results for the calculated ground state and excited state energies and charge radii as well as experimental data for comparison. The central values presented are obtained using the best fit values for the 2N and 3N coefficients. By this we mean the 2N coefficients that give the best fit for the scattering phase shifts and mixing angles, together with the 3N coefficients that give the best fit for the fitted nuclear binding energies shown in the main text. The quoted errors include all the statistical and systematic uncertainties such as computational uncertainties from Monte Carlo errors, infinite volume extrapolation, and infinite projection time extrapolation. For the energies and several charge radii where the history matching analysis is performed, the corresponding uncertainties from the history matching analysis are shown as a second set of quoted errors. These uncertainties

Table S9. Charge radii with history matching uncertainties. Charge radii of selected nuclei with uncertainties using history matching for WAVE 1 through WAVE 4. The central values and error bars are the mean values and standard deviations of the radii associated with points in the non-implausible volume.

Nuclei	WAVE 1	WAVE 2	WAVE 3	WAVE 4	Experiment (fm)
^{3}H	1.6938(55)	1.7053(35)	1.7136(61)	1.7074(91)	1.759
^{3}He	1.9040(126)	1.8954(77)	1.8933(205)	1.9054(267)	1.945
^{4}He	1.6127(163)	1.6647(116)	1.7136(171)	1.6919(181)	1.676
^{6}He	2.0482(146)	2.0037(61)	2.0055(80)	1.9987(96)	2.068(11)
^{6}Li	2.6759(96)	2.5772(112)	2.5362(130)	2.5487(161)	2.589(39)
^{7}Li	2.5384(297)	2.4620(145)	2.4376(252)	2.4228(352)	2.444(42)
^{9}Be	2.6956(234)	2.5509(136)	2.5093(150)	2.5158(255)	2.518
^{14}N	2.4679(271)	2.3952(254)	2.6185(291)	2.5557(383)	2.5579
^{20}Ne	3.0553(402)	2.8148(375)	3.0475(788)	3.0296(1002)	3.0053
^{28}Si	3.3879(920)	3.1620(604)	3.2142(926)	3.1316(665)	3.1223
^{40}Ca	3.5109(331)	3.3351(397)	3.5363(494)	3.4681(817)	3.4764

are the standard deviations of the binding energies and radii associated with points in the non-implausible volume. These errors include propagated uncertainties due to truncation of the effective field theory expansion. The errors on the charge radii grow for the heaviest nuclei, and the main sources of these errors are Monte Carlo statistics. Therefore, they can be reduced by using larger computer resources. In Table S11 we show the calculated pure neutron matter energies for various numbers of neutrons and box sizes as well as corresponding densities. Similarly, large scale calculations corresponding to high densities have larger errors which can be reduced by larger computer resources. In Table S12 we show the results for symmetric nuclear matter energies for various numbers of nucleons as well as corresponding densities. The large errors on the results with large numbers of nucleon are the statistical errors of Monte Carlo calculations and can be reduced by more computer power.

S18 Analysis of Three-Nucleon Interaction Contributions to the Binding Energies

In this section, we analyze the contributions of the 3N interactions and their varying degrees of importance by performing fits to the binding energies using $V_{c_E}^{(0)}$ and $V_{c_D}^{(0)}$ all possible subsets of the additional 3N interactions, $V_{c_E}^{(1)}$, $V_{c_E}^{(2)}$, $V_{c_D}^{(1)}$, $V_{c_D}^{(2)}$, $V_{c_E}^{(l)}$, and $V_{c_E}^{(t)}$. To end this, we use the best fit values for the 2N LECs, which give the scattering phase shifts and mixing angles shown by blue dashed lines in Fig. S10. We then perform least squares fits for some selected nuclear binding energies using subsets the 3N interactions. In this analysis we calculate the RMSD (root mean square deviation) over calculated nuclear binding energies shown in Fig. S11, and we use these results to assess the extent to which set of 3N interactions is the best for accurately describing some certain nuclear binding energies. The results are shown in Tables S13-S19, where we also present the energy differences $E_8^4 - 2E_4^2$, $E^6_{12,0_1^+} - 3E_4^2$, $E^6_{12,0_2^+} - 3E_4^2$, $E_{16}^8 - 4E_4^2$, $E_6^2 - E_4^2$, $E_9^4 - E_8^4$, $E_{13}^6 - E_{12}^6$, $E_{17}^8 - E_{16}^8$, $E_{10}^4 - E_8^4$, $E_{14}^6 - E_{12}^6$, and $E_{18}^8 - E_{16}^8$. Our notation is E_A^Z, where Z is the number of protons and A is the number of nucleons.

We start the RMSD analysis using the simpliest two 3N interactions, $V_{c_E}^{(0)}$ and $V_{c_D}^{(0)}$, and the nuclear binding energies for light nuclei whose structure are in the form of distinct geometrical configuration of α clusters. The results are shown in Table S13. As seen in Table S13, the RMSD of the binding energies per nucleon over all calculated nuclear binding energies is 1.236, and it corresponds to 17.3 % of the average nuclear binding energy per nucleon over all calculated nuclear binding energies $\frac{1}{N}\sum_{i=1}^{N} z_i/A_i = 7.01$, where N is the number of nuclei, z_i is the binding energy of the $i^{\rm th}$ nucleus and A_i is the number of nucleon of the $i^{\rm th}$ nucleus.

Now we use one more 3N interaction in addition to $V_{c_E}^{(0)}$ and $V_{c_D}^{(0)}$ and fit the nuclear binding energies for ^{4}He,^{8}Be,^{12}C, and ^{16}O. The results are given in Table S14. We compute the RMSD over all calculated nuclear binding energies, and we find the lowest value as 0.403 MeV which corresponds to 5.6 % of the average nuclear binding energy per nucleon and is obtained using the 3N interactions $V_{c_E}^{(0)}$, $V_{c_D}^{(0)}$ and $V_{c_E}^{(l)}$.

In the next analysis, we use two additional 3N interactions and fit the nuclear binding energies for ^{4}He,^{8}Be,^{12}C, and ^{16}O. We compute the RMSD over all calculated nuclear binding energies and the results are given in Table S15. By using $V_{c_E}^{(0)}$, $V_{c_D}^{(0)}$, $V_{c_E}^{(l)}$ and $V_{c_E}^{(t)}$ we find that the lowest value for the RMSD is 0.293 MeV, and this means that roughly we obtain the average nuclear binding energy per nucleon of all calculated nuclei with 4.1 % errors. The RMSD analysis and the results shown in Table S15 are consistent with results of history matching wathet-colored left-triangle points in FIG. S11.

After finding four 3N interactions which give a good description for light α-like nuclei, we include neutron-rich nuclei in RMSD analyses. We use three 3N interactions in addition to $V_{c_E}^{(0)}$ and $V_{c_D}^{(0)}$ and fit the nuclear binding energies for

^{3}H, ^{4}He, ^{7}Li, ^{8}Be, ^{9}Be, ^{10}Be, ^{10}B, ^{11}B, ^{12}C, ^{13}C, ^{14}C, ^{14}N, ^{15}N, ^{16}O, ^{17}O, and ^{18}O. We compute the RMSD over all calculated nuclear binding energies and the results are given in Table S16. We find that the lowest value for the RMSD is 0.131 MeV and is obtained by using $V_{c_E}^{(0)}$, $V_{c_D}^{(0)}$, $V_{c_E}^{(l)}$, $V_{c_E}^{(t)}$, and $V_{c_E}^{(1)}$, which means that the average nuclear binding energy per nucleon of all calculated nuclei is computed roughly with 1.83 % errors. The RMSD analysis and the results shown in Table S16.

Now we fit the nuclear binding energies for ^{3}H, ^{4}He, ^{7}Li, ^{8}Be, ^{9}Be, ^{10}Be, ^{10}B, ^{11}B, ^{12}C, ^{13}C, ^{14}C, ^{14}N, ^{15}N, ^{16}O, ^{17}O, and ^{18}O using four and five 3N interactions in addition to $V_{c_E}^{(0)}$ and $V_{c_D}^{(0)}$, and the results are shown in Table S17 and Table S18, respectively. The lowest value for the RMSD is found as 0.109(0.102) MeV when six(seven) 3N interactions are used in the fit, and this value corresponds to 1.54% (1.43%) of the average nuclear binding energy per nucleon.

Finally, we fit the nuclear binding energies for ^{3}H, ^{4}He, ^{7}Li, ^{8}Be, ^{9}Be, ^{10}Be, ^{10}B, ^{11}B, ^{12}C, ^{13}C, ^{14}C, ^{14}N, ^{15}N, ^{16}O, ^{17}O, ^{18}O, and ^{40}Ca using six 3N interactions in addition to $V_{c_E}^{(0)}$ and $V_{c_D}^{(0)}$, and the results are shown in Table S19. The lowest value for the RMSD is found as 0.079 MeV, which corresponds to 1.11 % of the average nuclear binding energy per nucleon.

References

1. Lee, D. Lattice simulations for few- and many-body systems. *Prog. Part. Nucl. Phys.* **63**, 117–154 (2009). 0804.3501.
2. Lähde, T. A. & Meißner, U.-G. *Nuclear Lattice Effective Field Theory: An introduction*, vol. 957 (Springer, 2019).
3. Borasoy, B., Epelbaum, E., Krebs, H., Lee, D. & Meißner, U.-G. Lattice Simulations for Light Nuclei: Chiral Effective Field Theory at Leading Order. *Eur. Phys. J. A* **31**, 105–123 (2007). nucl-th/0611087.
4. Meißner, U.-G. A new tool in nuclear physics: Nuclear lattice simulations. *Nucl. Phys. News.* **24**, 11–15 (2014). 1505.06997.
5. Meißner, U.-G. Structure of Nuclei from Lattice Simulations. *JPS Conf. Proc.* **6**, 010005 (2015).
6. Lee, D. Chiral Effective Field Theory after Thirty Years: Nuclear Lattice Simulations. *Few Body Syst.* **62**, 115 (2021). 2109.09582.
7. Krebs, H., Borasoy, B., Epelbaum, E., Lee, D. & Meißner, U.-G. Nuclear effective field theory on the lattice. *PoS* **LATTICE2008**, 023 (2008). 0810.0197.
8. Elhatisari, S. *et al.* Ab initio Calculations of the Isotopic Dependence of Nuclear Clustering. *Phys. Rev. Lett.* **119**, 222505 (2017). 1702.05177.
9. Epelbaum, E., Krebs, H., Lahde, T. A., Lee, D. & Meißner, U.-G. Structure and rotations of the Hoyle state. *Phys. Rev. Lett.* **109**, 252501 (2012). 1208.1328.
10. Lähde, T. A. *et al.* The Hoyle state in nuclear lattice effective field theory. *Pramana* **83**, 651–659 (2014). 1403.5451.
11. Shen, S. *et al.* Emergent geometry and duality in the carbon nucleus. *Nature Commun.* **14**, 2777 (2023). 2202.13596.
12. Epelbaum, E. *et al.* Ab Initio Calculation of the Spectrum and Structure of ^{16}O. *Phys. Rev. Lett.* **112**, 102501 (2014). 1312.7703.
13. Lähde, T. A. *et al.* Lattice Effective Field Theory for Medium-Mass Nuclei. *Phys. Lett. B* **732**, 110–115 (2014). 1311.0477.
14. Tichai, A., Roth, R. & Duguet, T. Many-Body Perturbation Theories for Finite Nuclei. *Frontiers in Physics* **8** (2020).
15. Frame, D. *et al.* Eigenvector continuation with subspace learning. *Phys. Rev. Lett.* **121**, 032501 (2018). 1711.07090.
16. Lähde, T. A. *et al.* Nuclear Lattice Simulations using Symmetry-Sign Extrapolation. *Eur. Phys. J. A* **51**, 92 (2015). 1502.06787.
17. Lu, B.-N. *et al.* Essential elements for nuclear binding. *Phys. Lett. B* **797**, 134863 (2019). 1812.10928.
18. Lu, B.-N. *et al.* *Ab Initio* Nuclear Thermodynamics. *Phys. Rev. Lett.* **125**, 192502 (2020). 1912.05105.
19. Reinert, P., Krebs, H. & Epelbaum, E. Semilocal momentum-space regularized chiral two-nucleon potentials up to fifth order. *Eur. Phys. J. A* **54**, 86 (2018). 1711.08821.
20. Fettes, N., Meißner, U.-G. & Steininger, S. Pion - nucleon scattering in chiral perturbation theory. 1. Isospin symmetric case. *Nucl. Phys. A* **640**, 199–234 (1998). hep-ph/9803266.
21. Li, N. *et al.* Neutron-proton scattering with lattice chiral effective field theory at next-to-next-to-next-to-leading order. *Phys. Rev. C* **98**, 044002 (2018). 1806.07994.
22. Friar, J. L., Huber, D. & van Kolck, U. Chiral symmetry and three nucleon forces. *Phys. Rev. C* **59**, 53–58 (1999). nucl-th/9809065.

23. Epelbaum, E. *et al.* Three nucleon forces from chiral effective field theory. *Phys. Rev. C* **66**, 064001 (2002). nucl-th/0208023.

24. Epelbaum, E., Krebs, H., Lee, D. & Meißner, U.-G. Lattice chiral effective field theory with three-body interactions at next-to-next-to-leading order. *Eur. Phys. J. A* **41**, 125–139 (2009). 0903.1666.

25. Stoks, V. G. J., Klomp, R. A. M., Rentmeester, M. C. M. & de Swart, J. J. Partial wave analaysis of all nucleon-nucleon scattering data below 350-MeV. *Phys. Rev. C* **48**, 792–815 (1993).

26. Lee, D. *et al.* Hidden Spin-Isospin Exchange Symmetry. *Phys. Rev. Lett.* **127**, 062501 (2021). 2010.09420.

27. Farhi, E., Goldstone, J., Gutmann, S. & Sipser, M. Quantum computation by adiabatic evolution (2000). quant-ph/0001106.

28. Elhatisari, S. *et al.* Ab initio alpha-alpha scattering. *Nature* **528**, 111 (2015). 1506.03513.

29. Householder, A. S. Unitary triangularization of a nonsymmetric matrix. *Journal of the ACM (JACM)* **5**, 339–342 (1958).

30. Givens, W. Computation of plain unitary rotations transforming a general matrix to triangular form. *Journal of the Society for Industrial and Applied Mathematics* **6**, 26–50 (1958).

31. Platter, L., Hammer, H. W. & Meißner, U.-G. On the correlation between the binding energies of the triton and the alpha-particle. *Phys. Lett. B* **607**, 254–258 (2005). nucl-th/0409040.

32. Rokash, A., Epelbaum, E., Krebs, H. & Lee, D. Effective forces between quantum bound states. *Phys. Rev. Lett.* **118**, 232502 (2017). 1612.08004.

33. Kanada-En'yo, Y. & Lee, D. Effective interactions between nuclear clusters. *Phys. Rev. C* **103**, 024318 (2021). 2008.01867.

34. Elhatisari, S. *et al.* Nuclear binding near a quantum phase transition. *Phys. Rev. Lett.* **117**, 132501 (2016). 1602.04539.

35. Coon, S. A. *et al.* Convergence properties of \it ab initio calculations of light nuclei in a harmonic oscillator basis. *Phys. Rev. C* **86**, 054002 (2012). 1205.3230.

36. Furnstahl, R. J., Hagen, G. & Papenbrock, T. Corrections to nuclear energies and radii in finite oscillator spaces. *Phys. Rev. C* **86**, 031301 (2012). 1207.6100.

37. Furnstahl, R. J., Papenbrock, T. & More, S. N. Systematic expansion for infrared oscillator basis extrapolations. *Phys. Rev. C* **89**, 044301 (2014). 1312.6876.

38. Coon, S. A. Infrared and ultraviolet cutoffs in variational calculations with a harmonic oscillator basis. In *International Conference on Nuclear Theory in the Supercomputing Era*, 171–188 (2013). 1303.6358.

39. Furnstahl, R. J., Hagen, G., Papenbrock, T. & Wendt, K. A. Infrared extrapolations for atomic nuclei. *J. Phys. G* **42**, 034032 (2015). 1408.0252.

40. Coon, S. A. & Kruse, M. K. G. Properties of infrared extrapolations in a harmonic oscillator basis. *Int. J. Mod. Phys. E* **25**, 1641011 (2016). 1408.0738.

41. Hoferichter, M., Ruiz de Elvira, J., Kubis, B. & Meißner, U.-G. Matching pion-nucleon Roy-Steiner equations to chiral perturbation theory. *Phys. Rev. Lett.* **115**, 192301 (2015). 1507.07552.

42. Bower, R. G., Goldstein, M. & Vernon, I. Galaxy formation: a Bayesian uncertainty analysis. *Bayesian Analysis* **5**, 619 – 669 (2010).

43. Vernon, I., Goldstein, M. & Bower, R. Galaxy Formation: Bayesian History Matching for the Observable Universe. *Statistical Science* **29**, 81 – 90 (2014).

44. Vernon, I. *et al.* Bayesian uncertainty analysis for complex systems biology models: emulation, global parameter searches and evaluation of gene functions. *BMC Syst. Biol.* **12**, 1–29 (2018).

45. Hu, B. *et al.* Ab initio predictions link the neutron skin of ^{208}Pb to nuclear forces. *Nature Phys.* **18**, 1196–1200 (2022). 2112.01125.

46. Epelbaum, E., Krebs, H. & Meißner, U.-G. Improved chiral nucleon-nucleon potential up to next-to-next-to-next-to-leading order. *Eur. Phys. J. A* **51**, 53 (2015). 1412.0142.

47. Epelbaum, E., Krebs, H. & Meißner, U.-G. Precision nucleon-nucleon potential at fifth order in the chiral expansion. *Phys. Rev. Lett.* **115**, 122301 (2015). 1412.4623.

48. Metropolis, N., Rosenbluth, A. W., Rosenbluth, M. N., Teller, A. H. & Teller, E. Equation of state calculations by fast computing machines. *The journal of chemical physics* **21**, 1087–1092 (1953).

49. Pukelsheim, F. The three sigma rule. *The American Statistician* **48**, 88–91 (1994).

50. Weinberg, S. Nuclear forces from chiral lagrangians. *Physics Letters B* **251**, 288–292 (1990).

51. Weinberg, S. Effective chiral lagrangians for nucleon-pion interactions and nuclear forces. *Nuclear Physics B* **363**, 3–18 (1991).

52. van Kolck, U. Few-nucleon forces from chiral lagrangians. *Phys. Rev. C* **49**, 2932–2941 (1994).

53. Epelbaum, E., Hammer, H.-W. & Meißner, U.-G. Modern Theory of Nuclear Forces. *Rev. Mod. Phys.* **81**, 1773–1825 (2009). 0811.1338.

54. Machleidt, R. & Entem, D. Chiral effective field theory and nuclear forces. *Physics Reports* **503**, 1–75 (2011).

55. Entem, D. R., Machleidt, R. & Nosyk, Y. High-quality two-nucleon potentials up to fifth order of the chiral expansion. *Phys. Rev. C* **96**, 024004 (2017).

56. Hammer, H.-W., König, S. & van Kolck, U. Nuclear effective field theory: Status and perspectives. *Rev. Mod. Phys.* **92**, 025004 (2020).

57. Epelbaum, E., Krebs, H. & Reinert, P. High-precision nuclear forces from chiral eft: State-of-the-art, challenges, and outlook. *Frontiers in Physics* **8** (2020).

58. Ekström, A. *et al.* Accurate nuclear radii and binding energies from a chiral interaction. *Phys. Rev. C* **91**, 051301 (2015).

59. Navrátil, P., Gueorguiev, V. G., Vary, J. P., Ormand, W. E. & Nogga, A. Structure of $a = 10 - -13$ nuclei with two- plus three-nucleon interactions from chiral effective field theory. *Phys. Rev. Lett.* **99**, 042501 (2007).

60. Roth, R., Langhammer, J., Calci, A., Binder, S. & Navrátil, P. Similarity-transformed chiral $nn + 3n$ interactions for the ab initio description of ^{12}**C** and ^{16}**O**. *Phys. Rev. Lett.* **107**, 072501 (2011).

61. Hebeler, K. Three-nucleon forces: Implementation and applications to atomic nuclei and dense matter. *Phys. Rept.* **890**, 1–116 (2021). 2002.09548.

62. Bogner, S., Furnstahl, R. & Schwenk, A. From low-momentum interactions to nuclear structure. *Progress in Particle and Nuclear Physics* **65**, 94–147 (2010).

63. Barrett, B. R., Navrátil, P. & Vary, J. P. Ab initio no core shell model. *Progress in Particle and Nuclear Physics* **69**, 131–181 (2013).

64. Roth, R., Calci, A., Langhammer, J. & Binder, S. Evolved chiral $nn + 3n$ hamiltonians for ab initio nuclear structure calculations. *Phys. Rev. C* **90**, 024325 (2014).

65. Jurgenson, E. D. *et al.* Structure of p-shell nuclei using three-nucleon interactions evolved with the similarity renormalization group. *Phys. Rev. C* **87**, 054312 (2013).

66. Maris, P. *et al.* Nuclear properties with semilocal momentum-space regularized chiral interactions beyond N2LO (2022). 2206.13303.

67. Dytrych, T. *et al.* Physics of nuclei: Key role of an emergent symmetry. *Phys. Rev. Lett.* **124**, 042501 (2020).

68. Dreyfuss, A. C. *et al.* Clustering and α-capture reaction rate from ab initio symmetry-adapted descriptions of ^{20}Ne. *Phys. Rev. C* **102**, 044608 (2020).

69. Carlson, J. *et al.* Quantum monte carlo methods for nuclear physics. *Rev. Mod. Phys.* **87**, 1067–1118 (2015).

70. Pastore, S. *et al.* Quantum Monte Carlo calculations of weak transitions in $A = 6$–10 nuclei. *Phys. Rev. C* **97**, 022501 (2018). 1709.03592.

71. Lynn, J., Tews, I., Gandolfi, S. & Lovato, A. Quantum monte carlo methods in nuclear physics: Recent advances. *Annual Review of Nuclear and Particle Science* **69**, 279–305 (2019). https://doi.org/10.1146/annurev-nucl-101918-023600.

72. Gandolfi, S., Lonardoni, D., Lovato, A. & Piarulli, M. Atomic nuclei from quantum Monte Carlo calculations with chiral EFT interactions. *Front. in Phys.* **8**, 117 (2020). 2001.01374.

73. Schiavilla, R. *et al.* Two- and three-nucleon contact interactions and ground-state energies of light- and medium-mass nuclei. *Phys. Rev. C* **103**, 054003 (2021). 2102.02327.

74. Somà, V. Self-consistent Green's function theory for atomic nuclei. *Front. in Phys.* **8**, 340 (2020). 2003.11321.

75. Roth, R. & Langhammer, J. Padé-resummed high-order perturbation theory for nuclear structure calculations. *Physics Letters B* **683**, 272–277 (2010). URL https://www.sciencedirect.com/science/article/pii/S037026930901507X.

76. Tichai, A., Langhammer, J., Binder, S. & Roth, R. Hartree-fock many-body perturbation theory for nuclear ground-states. *Physics Letters B* **756**, 283–288 (2016). URL https://www.sciencedirect.com/science/article/pii/S0370269316002008.

77. Hagen, G., Papenbrock, T., Hjorth-Jensen, M. & Dean, D. J. Coupled-cluster computations of atomic nuclei. *Rept. Prog. Phys.* **77**, 096302 (2014). 1312.7872.

78. Hergert, H., Bogner, S., Morris, T., Schwenk, A. & Tsukiyama, K. The in-medium similarity renormalization group: A novel ab initio method for nuclei. *Physics Reports* **621**, 165–222 (2016). Memorial Volume in Honor of Gerald E. Brown.

79. Abe, T. *et al.* Ground-state properties of light 4n self-conjugate nuclei in ab initio no-core Monte Carlo shell model calculations with nonlocal NN interactions. *Phys. Rev. C* **104**, 054315 (2021). 2106.15114.

80. Otsuka, T. *et al.* α-Clustering in atomic nuclei from first principles with statistical learning and the Hoyle state character. *Nature Commun.* **13**, 2234 (2022).

81. Hjorth-Jensen, M., Kuo, T. T. & Osnes, E. Realistic effective interactions for nuclear systems. *Physics Reports* **261**, 125–270 (1995).

82. Coraggio, L., Covello, A., Gargano, A., Itaco, N. & Kuo, T. Shell-model calculations and realistic effective interactions. *Progress in Particle and Nuclear Physics* **62**, 135–182 (2009).

83. Tichai, A. *et al.* Bogoliubov many-body perturbation theory for open-shell nuclei. *Physics Letters B* **786**, 195–200 (2018).

84. Bogner, S. K. *et al.* Nonperturbative shell-model interactions from the in-medium similarity renormalization group. *Phys. Rev. Lett.* **113**, 142501 (2014).

85. Stroberg, S. R. *et al.* A nucleus-dependent valence-space approach to nuclear structure. *Phys. Rev. Lett.* **118**, 032502 (2017). 1607.03229.

86. Jansen, G. R., Engel, J., Hagen, G., Navrátil, P. & Signoracci, A. Ab initio coupled-cluster effective interactions for the shell model: Application to neutron-rich oxygen and carbon isotopes. *Phys. Rev. Lett.* **113**, 142502 (2014).

87. Sun, Z. H., Morris, T. D., Hagen, G., Jansen, G. R. & Papenbrock, T. Shell-model coupled-cluster method for open-shell nuclei. *Phys. Rev. C* **98**, 054320 (2018).

88. Navrátil, P., Quaglioni, S., Hupin, G., Romero-Redondo, C. & Calci, A. Unified ab initio approaches to nuclear structure and reactions. *Physica Scripta* **91**, 053002 (2016).

89. Hagen, G., Hjorth-Jensen, M., Jansen, G. R., Machleidt, R. & Papenbrock, T. Continuum effects and three-nucleon forces in neutron-rich oxygen isotopes. *Phys. Rev. Lett.* **108**, 242501 (2012).

90. Papadimitriou, G., Rotureau, J., Michel, N., Płoszajczak, M. & Barrett, B. R. Ab initio no-core gamow shell model calculations with realistic interactions. *Phys. Rev. C* **88**, 044318 (2013).

91. Li, J. G., Michel, N., Hu, B. S., Zuo, W. & Xu, F. R. Ab initio no-core gamow shell-model calculations of multineutron systems. *Phys. Rev. C* **100**, 054313 (2019).

92. Hu, B. S., Wu, Q., Sun, Z. H. & Xu, F. R. Ab initio gamow in-medium similarity renormalization group with resonance and continuum. *Phys. Rev. C* **99**, 061302 (2019).

93. Sun, Z. *et al.* Resonance and continuum gamow shell model with realistic nuclear forces. *Physics Letters B* **769**, 227–232 (2017).

94. Ma, Y. *et al.* Chiral three-nucleon force and continuum for dripline nuclei and beyond. *Physics Letters B* **802**, 135257 (2020).

95. Ekström, A. *et al.* What is ab initio in nuclear theory? *Front. Phys.* **11**, 1129094 (2023). 2212.11064.

96. Machleidt, R. What is ab initio? *Few Body Syst.* **64**, 77 (2023). 2307.06416.

97. Hupin, G. *et al.* Ab initio many-body calculations of nucleon-4He scattering with three-nucleon forces. *Phys. Rev. C* **88**, 054622 (2013). 1308.2700.

98. Lynn, J. E. *et al.* Chiral Three-Nucleon Interactions in Light Nuclei, Neutron-α Scattering, and Neutron Matter. *Phys. Rev. Lett.* **116**, 062501 (2016). 1509.03470.

99. Bertulani, C. A., Hammer, H. W. & Van Kolck, U. Effective field theory for halo nuclei. *Nucl. Phys. A* **712**, 37–58 (2002). nucl-th/0205063.

100. Higa, R., Hammer, H. W. & van Kolck, U. alpha alpha Scattering in Halo Effective Field Theory. *Nucl. Phys. A* **809**, 171–188 (2008). 0802.3426.

101. Rotureau, J. & van Kolck, U. Effective Field Theory and the Gamow Shell Model: The ^{6}He Halo Nucleus. *Few Body Syst.* **54**, 725–735 (2013). 1201.3351.

102. Hammer, H. W., Ji, C. & Phillips, D. R. Effective field theory description of halo nuclei. *J. Phys. G* **44**, 103002 (2017). 1702.08605.

103. Pohl, R. *et al.* The size of the proton. *Nature* **466**, 213–216 (2010).

104. Lin, Y.-H., Hammer, H.-W. & Meißner, U.-G. New Insights into the Nucleon's Electromagnetic Structure. *Phys. Rev. Lett.* **128**, 052002 (2022). 2109.12961.

105. Filin, A. A. *et al.* High-accuracy calculation of the deuteron charge and quadrupole form factors in chiral effective field theory. *Phys. Rev. C* **103**, 024313 (2021). 2009.08911.

Table S10. Binding energies and charge radii of selected nuclei. We show lattice results at N3LO and the comparison with experimental data. For all the calculated energies and radii, the quoted central values are computed using 2N LECs set to the best fit values for the scattering phase shifts and mixing angles, together with the 3N coefficients that produce the best fit for the fitted binding energies shown in the main text. The first error is the estimate of the computational errors. Where listed, the second error bar corresponds to the uncertainty due to the chiral interactions, as estimated from the history matching analysis. Otherwise, we have written "NA" for "not available". For both error bars, the last digit of the error bar corresponds to the same digit place as the last digit of the central value.

Nuclei	B (MeV)	Experiment (MeV)	R_c (fm)	Experiment (fm)
^{2}H	2.2102	2.2246	2.140	2.140
^{3}H	8.35(17)(6)	8.48	1.7132(22)(91)	1.759
^{3}He	7.64(17)(4)	7.72	1.9013(84)(264)	1.945
^{4}He	28.24(9)(17)	28.3	1.7034(29)(181)	1.676
^{6}He	29.04(9)(15)	29.27	1.9961(228)(96)	2.068(11)
^{6}Li	32.82(9)(13)	31.99	2.5343(248)(161)	2.589(39)
^{7}Li	39.61(8)(11)	39.24	2.4277(191)(352)	2.444(42)
^{8}Be	56.73(21)(31)	56.5		
^{9}Be	57.59(20)(24)	58.17	2.5104(337)(255)	2.518
^{10}Be	63.72(14)(16)	64.97		
^{10}B	64.46(56)(53)	64.75	2.4707(348)(NA)	2.4278
^{11}B	75.38(13)(83)	76.2	2.4311(194)(NA)	2.4059
$^{12}C_{0_1^+}$	92.36(61)(125)	92.16	2.4759(121)(NA)	2.470
$^{12}C_{0_2^+}$	84.88(60)(167)	84.51		
$^{12}C_{2_1^+}$	87.58(70)(123)	87.72		
^{13}C	97.07(26)(55)	97.11	2.5028(408)(NA)	2.4614
^{14}C	104.87(51)(59)	105.28	2.5438(396)(NA)	2.504
^{14}N	106.25(55)(83)	104.66	2.5608(216)(383)	2.5579
^{15}N	115.29(22)(39)	115.49	2.6425(471)(NA)	2.6061
^{16}O	129.99(19)(48)	127.62	2.6801(180)(NA)	2.701
^{17}O	132.47(26)(41)	131.76	2.6707(756)(NA)	2.695
^{18}O	140.37(21)(39)	139.81	2.7478(498)(NA)	2.775
^{20}O	151.90(101)(56)	151.37		
^{22}O	160.70(110)(85)	162.03		
^{24}O	166.88(71)(104)	168.38		
^{18}F	135.52(44)(54)	137.37		
^{20}Ne	164.57(32)(44)	160.65	2.9851(319)(1002)	3.0053
^{24}Mg	195.96(85)(177)	198.26	3.0486(269)(NA)	3.0568
^{28}Si	234.16(50)(195)	236.54	3.0856(227)(665)	3.1223
^{32}S	266.79(69)(185)	271.78	3.2339(703)(NA)	3.2608
^{36}Ar	299.68(99)(357)	306.72	3.4365(1075)(NA)	3.3902
^{40}Ca	337.71(134)(170)	342.05	3.4892(875)(817)	3.4764
^{50}Cr	426.65(166)(464)	435.05	3.6369(593)(NA)	3.6588(65)
^{58}Ni	502.02(155)(608)	506.46	3.7754(740)(NA)	3.7757(20)

Table S11. Neutron matter results. Pure neutron matter energies using various number of neutrons and box sizes.

$A(=N)$	L (fm)	ρ (fm^{-3})	E (MeV)
14	6.58	0.0492	102.9(1)
14	7.89	0.0285	66.84(92)
14	9.21	0.0179	51.41(76)
14	10.5	0.0120	41.41(85)
14	11.8	0.0084	34.11(65)
14	13.2	0.0062	28.71(50)
28	6.58	0.0984	341.5(99)
28	7.89	0.0570	220.8(64)
28	9.21	0.0359	169.9(35)
28	10.5	0.0240	136.0(24)
28	11.8	0.0169	112.8(21)
42	6.58	0.1477	607.4(70)
42	7.89	0.0855	359.9(42)
42	9.21	0.0538	267.3(43)
42	10.5	0.0361	211.1(10)
42	11.8	0.0253	175.7(10)
42	13.2	0.0185	149.6(22)
66	6.58	0.2320	1750(7)
66	7.89	0.1343	928.4(17)
66	9.21	0.0846	613.1(44)
66	10.5	0.0567	464.0(66)
66	11.8	0.0398	373(19)
80	6.58	0.2812	2429(10)
80	7.89	0.1628	1535(7)
80	9.21	0.1025	999(8)

Table S12. Symmetric nuclear matter results. Symmetric nuclear matter energies for various number of nucleons with box length $L = 9.21$ fm.

$A(=2N=2Z)$	L (fm)	ρ (fm^{-3})	E at N3LO [2NFs only] (MeV)	E at N3LO [2NFs+3NFs] (MeV)
12	9.21	0.0154	−101.4(2)	−102.4(7)
16	9.21	0.0205	−137.3(3)	−164(2)
24	9.21	0.0308	−219.9(1)	−281(3)
36	9.21	0.0461	−328(1)	−456(2)
48	9.21	0.0615	−453(1)	−697(4)
60	9.21	0.0768	−569(2)	−851(6)
72	9.21	0.0922	−699(1)	−1067(5)
84	9.21	0.1076	−830(2)	−1328(5)
96	9.21	0.1230	−965(3)	−1642(6)
112	9.21	0.1435	−1090(2)	−1945(8)
128	9.21	0.1640	−1060(2)	−2078(7)
144	9.21	0.1845	−963(7)	−2150(43)
160	9.21	0.2050	−850(39)	−2437(317)

Table S13. Binding energy errors with $V_{c_E}^{(0)}$ and $V_{c_D}^{(0)}$ only. Results for the RMSD (root mean square deviation) over all calculated nuclear binding energies by using the simplest 3N interactions $V_{c_E}^{(0)}$ and $V_{c_D}^{(0)}$ to fit the nuclear binding energies for ^{4}He,^{8}Be,^{12}C, and ^{16}O. All energies are measured in MeV.

3N interactions in addition to $V_{c_E}^{(0)}, V_{c_D}^{(0)}$	RMSD B/A	$E_8^4 - 2E_4^2$	$E_{12,0_1^+}^6 - 3E_4^2$<br>$E_{12,0_2^+}^6 - 3E_4^2$	$E_{16}^8 - 4E_4^2$	$E_6^2 - E_4^2$	$E_9^4 - E_8^4$<br>$E_{10}^4 - E_8^4$	$E_{13}^6 - E_{12}^6$<br>$E_{14}^6 - E_{12}^6$	$E_{17}^8 - E_{16}^8$<br>$E_{18}^8 - E_{16}^8$
		0.10	−7.26<br>0.39	−14.42	−0.97	−1.67<br>−8.47	−4.95<br>−13.12	−4.14<br>−12.19
none	1.236	−0.42(50)	−0.86(82)<br>−0.19(92)	7.64(76)	0.23(19)	1.47(48)<br>−1.04(69)	4.98(99)<br>1.33(86)	−0.32(58)<br>−4.34(59)

Table S14. Binding energy errors when including one additional 3N interaction. Results for the RMSD over all calculated nuclear binding energies when using one additional 3N interaction as well as $V_{c_E}^{(0)}$ and $V_{c_D}^{(0)}$ to fit the nuclear binding energies for ^{4}He,^{8}Be,^{12}C, and ^{16}O. All energies are measured in MeV.

3N interactions in addition to	RMSD B/A	$E_8^4 - 2E_4^2$	$E_{12,0_1^+}^6 - 3E_4^2$<br>$E_{12,0_2^+}^6 - 3E_4^2$	$E_{16}^8 - 4E_4^2$	$E_6^2 - E_4^2$	$E_9^4 - E_8^4$<br>$E_{10}^4 - E_8^4$	$E_{13}^6 - E_{12}^6$<br>$E_{14}^6 - E_{12}^6$	$E_{17}^8 - E_{16}^8$<br>$E_{18}^8 - E_{16}^8$
$V_{c_E}^{(0)}, V_{c_D}^{(0)}$		0.10	−7.26<br>0.39	−14.42	−0.97	−1.67<br>−8.47	−4.95<br>−13.12	−4.14<br>−12.19
$V_{c_E}^{(l)}$	0.403	−0.57(51)	−6.16(82)<br>−1.15(97)	−15.17(77)	−0.17(19)	0.34(50)<br>−3.23(71)	0.85(98)<br>−5.97(86)	−1.88(59)<br>−7.10(60)
$V_{c_E}^{(2)}$	0.410	−0.53(52)	−5.58(82)<br>−0.46(109)	−15.07(76)	−0.09(19)	0.46(51)<br>−3.01(68)	0.78(98)<br>−6.19(86)	−1.86(58)<br>−6.95(59)
$V_{c_E}^{(t)}$	0.415	−0.30(50)	−4.04(82)<br>0.45(96)	−14.45(77)	0.07(19)	0.69(50)<br>−2.49(69)	0.34(98)<br>−6.65(86)	−1.85(59)<br>−6.72(60)
$V_{c_D}^{(2)}$	0.419	−0.30(56)	−2.28(82)<br>3.20(121)	−13.05(76)	−0.12(19)	0.65(52)<br>−3.19(59)	−1.97(98)<br>−8.90(85)	−1.95(58)<br>−6.73(59)
$V_{c_E}^{(1)}$	0.465	−0.72(57)	−5.36(82)<br>−0.37(100)	−14.96(76)	−0.18(19)	0.46(54)<br>−3.25(60)	1.66(98)<br>−5.38(89)	−1.62(58)<br>−6.72(59)
$V_{c_D}^{(1)}$	0.542	−0.09(63)	2.06(82)<br>7.30(175)	−7.17(76)	0.01(19)	1.22(63)<br>−2.84(87)	−2.49(98)<br>−8.81(86)	−1.82(58)<br>−6.29(59)

Table S15. Binding energy errors when including two additional 3N interactions. Results for the RMSD over all calculated nuclear binding energies by using two additional 3N interactions as well as $V_{c_E}^{(0)}$ and $V_{c_D}^{(0)}$ to fit the nuclear binding energies for ^{4}He,^{8}Be,^{12}C, and ^{16}O. All energies are measured in MeV.

3N interactions in addition to $V_{c_E}^{(0)}, V_{c_D}^{(0)}$	RMSD B/A	$E_8^4 - 2E_4^2$	$E_{12,0_1^+}^6 - 3E_4^2$<br>$E_{12,0_2^+}^6 - 3E_4^2$	$E_{16}^8 - 4E_4^2$	$E_6^2 - E_4^2$	$E_9^4 - E_8^4$<br>$E_{10}^4 - E_8^4$	$E_{13}^6 - E_{12}^6$<br>$E_{14}^6 - E_{12}^6$	$E_{17}^8 - E_{16}^8$<br>$E_{18}^8 - E_{16}^8$
		0.10	−7.26<br>0.39	−14.42	−0.97	−1.67<br>−8.47	−4.95<br>−13.12	−4.14<br>−12.19
$V_{c_E}^{(l)}, V_{c_E}^{(t)}$	0.293	−0.75(49)	−7.66(85)<br>−1.09(90)	−15.25(76)	−0.89(19)	−0.56(48)<br>−6.14(71)	−1.83(100)<br>−8.19(88)	−2.50(58)<br>−8.66(59)
$V_{c_E}^{(2)}, V_{c_E}^{(l)}$	0.342	−0.29(60)	−7.49(84)<br>−4.05(283)	−14.80(81)	−0.92(19)	−0.76(58)<br>−6.08(66)	−2.19(100)<br>−7.63(89)	−2.56(67)<br>−8.80(66)
$V_{c_D}^{(2)}, V_{c_E}^{(l)}$	0.362	−0.25(58)	−7.47(82)<br>−4.34(155)	−14.76(78)	−0.08(19)	0.15(55)<br>−3.36(65)	2.57(98)<br>−3.83(86)	−2.13(60)<br>−7.86(61)
$V_{c_D}^{(1)}, V_{c_E}^{(l)}$	0.384	−0.20(59)	−7.40(82)<br>−4.37(187)	−14.72(77)	−0.11(19)	0.05(59)<br>−3.25(87)	1.05(98)<br>−5.52(86)	−2.03(59)<br>−7.41(60)
$V_{c_D}^{(1)}, V_{c_D}^{(2)}$	0.405	−0.24(49)	−4.34(82)<br>0.27(95)	−14.29(76)	−0.14(19)	0.32(48)<br>−3.23(72)	−1.87(99)<br>−8.84(85)	−1.96(58)<br>−6.80(59)
$V_{c_E}^{(2)}, V_{c_E}^{(t)}$	0.418	−0.89(54)	−7.66(82)<br>−1.49(134)	−15.38(76)	−0.34(19)	0.15(52)<br>−3.79(62)	1.33(98)<br>−5.54(85)	−1.87(58)<br>−7.32(59)
$V_{c_E}^{(2)}, V_{c_D}^{(1)}$	0.423	−0.29(54)	−7.45(82)<br>−4.14(139)	−14.80(77)	0.02(19)	0.23(56)<br>−2.72(90)	1.85(98)<br>−4.95(86)	−1.87(59)<br>−7.08(60)
$V_{c_E}^{(1)}, V_{c_E}^{(2)}$	0.423	−0.60(53)	−5.73(82)<br>−0.58(108)	−15.04(76)	−0.10(19)	0.47(51)<br>−2.99(68)	1.08(98)<br>−5.89(86)	−1.81(58)<br>−6.91(59)
$V_{c_E}^{(2)}, V_{c_D}^{(2)}$	0.423	−0.23(57)	−7.46(82)<br>−4.48(116)	−14.74(77)	0.24(19)	0.48(54)<br>−2.33(64)	4.38(98)<br>−2.40(85)	−1.97(59)<br>−7.50(60)
$V_{c_E}^{(1)}, V_{c_E}^{(l)}$	0.427	−0.67(52)	−6.32(82)<br>−1.29(99)	−15.24(77)	−0.18(19)	0.36(52)<br>−3.21(69)	1.37(98)<br>−5.50(86)	−1.78(59)<br>−6.98(60)
$V_{c_D}^{(1)}, V_{c_E}^{(t)}$	0.430	0.10(59)	−5.04(82)<br>−2.90(178)	−14.18(78)	0.31(19)	0.61(64)<br>−1.79(109)	1.26(99)<br>−5.61(86)	−1.84(61)<br>−6.65(62)
$V_{c_E}^{(1)}, V_{c_D}^{(1)}$	0.525	−0.71(49)	−7.65(83)<br>−4.51(161)	−15.19(77)	−0.18(19)	0.14(47)<br>−3.26(57)	3.87(99)<br>−3.13(97)	−1.39(58)<br>−6.65(60)
$V_{c_E}^{(1)}, V_{c_E}^{(t)}$	0.538	−1.23(73)	−6.36(84)<br>−0.66(104)	−15.48(76)	−0.52(19)	0.18(71)<br>−4.40(91)	2.85(100)<br>−4.29(99)	−1.37(58)<br>−6.77(59)
$V_{c_E}^{(1)}, V_{c_D}^{(2)}$	0.545	−1.06(59)	−7.80(82)<br>−3.45(90)	−15.50(77)	−0.21(19)	0.33(57)<br>−3.21(63)	4.91(98)<br>−2.05(96)	−1.28(58)<br>−6.60(60)
$V_{c_D}^{(2)}, V_{c_E}^{(t)}$	0.555	0.40(77)	−5.45(84)<br>−6.17(225)	−13.95(84)	1.01(20)	1.28(88)<br>0.48(147)	6.95(100)<br>0.23(90)	−1.59(70)<br>−6.37(68)

Table S16. Binding energy errors when including three additional 3N interactions. Results for the RMSD over all calculated nuclear binding energies by using three additional 3N interactions as well as $V_{c_E}^{(0)}$ and $V_{c_D}^{(0)}$ to fit the nuclear binding energies for ^{3}H, ^{4}He, ^{7}Li, ^{8}Be, ^{9}Be, ^{10}Be, ^{10}B, ^{11}B, ^{12}C, ^{13}C, ^{14}C, ^{14}N, ^{15}N, ^{16}O, ^{17}O, and ^{18}O. All energies are measured in MeV.

3N interactions in addition to $V_{c_E}^{(0)}, V_{c_D}^{(0)}$	RMSD B/A	$E_8^4-2E_4^2$	$E^6_{12,0_1^+}-3E_4^2$ $E^6_{12,0_2^+}-3E_4^2$	$E_{16}^8-4E_4^2$	$E_6^2-E_4^2$	$E_9^4-E_8^4$ $E_{10}^4-E_8^4$	$E_{13}^6-E_{12}^6$ $E_{14}^6-E_{12}^6$	$E_{17}^8-E_{16}^8$ $E_{18}^8-E_{16}^8$
		0.10	−7.26	−14.42	−0.97	−1.67	−4.95	−4.14
			0.39			−8.47	−13.12	−12.19
$V_{c_E}^{(1)}, V_{c_E}^{(l)}$	0.131	−0.52(51)	−8.47(85)	−17.40(76)	−0.98(19)	−0.86(50)	−3.97(100)	−3.07(58)
$V_{c_E}^{(t)}$			−1.30(97)			−6.69(66)	−10.38(92)	−9.56(59)
$V_{c_E}^{(1)}, V_{c_E}^{(2)}$	0.169	0.13(61)	−5.55(82)	−16.61(78)	−0.25(19)	−0.02(61)	−3.96(98)	−2.95(61)
$V_{c_E}^{(l)}$			−0.44(107)			−4.02(90)	−10.56(92)	−8.57(61)
$V_{c_E}^{(1)}, V_{c_D}^{(2)}$	0.184	0.11(61)	−5.93(82)	−16.52(78)	−0.15(19)	0.07(63)	−3.47(99)	−2.92(60)
$V_{c_E}^{(l)}$			−0.71(91)			−3.59(102)	−10.17(95)	−8.46(61)
$V_{c_E}^{(1)}, V_{c_D}^{(1)}$	0.188	0.22(70)	−6.09(82)	−16.34(78)	−0.13(19)	0.01(72)	−3.58(99)	−2.96(61)
$V_{c_E}^{(l)}$			−1.38(117)			−3.59(112)	−10.20(95)	−8.52(61)
$V_{c_E}^{(2)}, V_{c_D}^{(1)}$	0.204	−0.12(55)	−5.83(83)	−17.25(78)	−0.39(19)	−0.16(52)	−2.01(99)	−2.54(61)
$V_{c_E}^{(l)}$			−1.60(185)			−4.50(59)	−8.88(87)	−8.22(61)
$V_{c_E}^{(1)}, V_{c_E}^{(2)}$	0.215	−1.32(53)	−11.54(82)	−18.00(77)	−0.81(19)	−0.59(49)	0.24(98)	−2.43(61)
$V_{c_E}^{(t)}$			−2.80(202)			−5.52(55)	−6.86(90)	−8.71(61)
$V_{c_E}^{(2)}, V_{c_E}^{(l)}$	0.217	−1.21(51)	−10.95(85)	−18.60(77)	−1.20(19)	−1.05(52)	−1.30(101)	−2.65(59)
$V_{c_E}^{(t)}$			−2.77(95)			−7.18(80)	−8.02(89)	−9.35(60)
$V_{c_E}^{(2)}, V_{c_D}^{(2)}$	0.230	−0.58(53)	−5.69(82)	−18.51(77)	−0.60(19)	−0.13(50)	−2.95(99)	−2.36(60)
$V_{c_E}^{(l)}$			0.51(89)			−4.78(62)	−10.06(86)	−7.84(60)
$V_{c_D}^{(1)}, V_{c_E}^{(l)}$	0.236	−1.42(70)	−9.45(86)	−19.01(77)	−1.41(19)	−0.97(74)	−2.68(102)	−2.72(59)
$V_{c_E}^{(t)}$			0.47(194)			−7.81(119)	−9.40(90)	−9.44(60)
$V_{c_E}^{(1)}, V_{c_E}^{(2)}$	0.240	−0.54(52)	−5.42(82)	−17.28(76)	−0.21(19)	0.31(50)	−1.09(98)	−2.17(58)
$V_{c_D}^{(2)}$			0.56(122)			−3.46(64)	−8.53(85)	−7.37(59)
$V_{c_D}^{(1)}, V_{c_D}^{(2)}$	0.249	−0.40(52)	−7.48(82)	−17.86(77)	−0.26(19)	−0.08(52)	−0.38(99)	−2.18(59)
$V_{c_E}^{(l)}$			−3.13(134)			−3.69(80)	−7.67(86)	−7.58(60)
$V_{c_E}^{(1)}, V_{c_E}^{(2)}$	0.250	−0.28(55)	−7.37(82)	−16.83(77)	−0.03(19)	0.16(56)	0.11(99)	−2.29(59)
$V_{c_D}^{(1)}$			−2.96(98)			−2.98(92)	−7.08(86)	−7.65(60)
$V_{c_E}^{(2)}, V_{c_D}^{(1)}$	0.252	−0.43(49)	−5.99(82)	−17.49(76)	−0.26(19)	0.08(49)	−1.66(99)	−2.13(58)
$V_{c_D}^{(2)}$			−0.92(96)			−3.60(76)	−9.14(85)	−7.25(59)
$V_{c_D}^{(2)}, V_{c_E}^{(l)}$	0.255	−1.24(62)	−8.95(88)	−18.75(77)	−1.47(19)	−1.14(67)	−4.83(103)	−2.82(60)
$V_{c_E}^{(t)}$			0.73(128)			−8.11(109)	−11.61(91)	−9.37(60)
$V_{c_E}^{(1)}, V_{c_D}^{(1)}$	0.263	−0.51(49)	−6.28(82)	−17.75(76)	−0.29(19)	0.05(49)	−1.27(99)	−2.06(58)
$V_{c_D}^{(2)}$			−1.21(95)			−3.66(73)	−8.78(86)	−7.21(59)
$V_{c_E}^{(2)}, V_{c_D}^{(2)}$	0.264	−1.54(70)	−10.22(83)	−18.31(79)	−1.07(19)	−0.67(71)	−0.88(99)	−2.31(63)
$V_{c_E}^{(t)}$			−0.60(232)			−6.42(93)	−8.19(88)	−8.54(62)
$V_{c_E}^{(2)}, V_{c_D}^{(1)}$	0.265	−1.65(66)	−11.56(83)	−18.64(78)	−0.99(19)	−0.65(65)	1.05(99)	−2.20(62)
$V_{c_E}^{(t)}$			−2.36(229)			−6.08(81)	−6.21(87)	−8.56(62)
$V_{c_D}^{(1)}, V_{c_D}^{(2)}$	0.267	−0.63(58)	−4.90(82)	−17.43(76)	−0.59(19)	−0.09(55)	−4.51(99)	−2.23(58)
$V_{c_E}^{(t)}$			2.13(108)			−4.66(64)	−12.07(85)	−7.31(59)
$V_{c_E}^{(1)}, V_{c_D}^{(2)}$	0.320	−1.00(75)	−4.62(85)	−17.46(76)	−0.64(19)	0.07(75)	−1.05(100)	−1.90(58)
$V_{c_E}^{(t)}$			2.40(132)			−4.98(103)	−8.75(93)	−7.28(59)
$V_{c_E}^{(1)}, V_{c_D}^{(1)}$	0.343	−1.16(63)	−7.97(82)	−18.56(76)	−0.47(19)	−0.00(60)	2.78(98)	−1.61(58)
$V_{c_E}^{(t)}$			−2.36(98)			−4.16(65)	−4.84(94)	−7.15(59)

Table S17. Binding energy errors when including four additional 3N interactions. Results for the RMSD over all calculated nuclear binding energies by using four additional 3N interactions as well as $V_{c_E}^{(0)}$ and $V_{c_D}^{(0)}$ to fit the nuclear binding energies for ^{3}H, ^{4}He, ^{7}Li, ^{8}Be, ^{9}Be, ^{10}Be, ^{10}B, ^{11}B, ^{12}C, ^{13}C, ^{14}C, ^{14}N, ^{15}N, ^{16}O, ^{17}O, and ^{18}O. All energies are measured in MeV.

3N interactions in addition to $V_{c_E}^{(0)}, V_{c_D}^{(0)}$	RMSD B/A	$E_8^4 - 2E_4^2$<br>0.10	$E^6_{12,0_1^+} - 3E_4^2$<br>$E^6_{12,0_2^+} - 3E_4^2$<br>−7.26<br>0.39	$E_{16}^8 - 4E_4^2$<br>−14.42	$E_6^2 - E_4^2$<br>−0.97	$E_9^4 - E_8^4$<br>$E_{10}^4 - E_8^4$<br>−1.67<br>−8.47	$E_{13}^6 - E_{12}^6$<br>$E_{14}^6 - E_{12}^6$<br>−4.95<br>−13.12	$E_{17}^8 - E_{16}^8$<br>$E_{18}^8 - E_{16}^8$<br>−4.14<br>−12.19
$V_{c_E}^{(1)}, V_{c_E}^{(2)}$<br>$V_{c_E}^{(l)}, V_{c_E}^{(t)}$	0.109	−0.67(51)	−9.63(84)<br>−1.34(92)	−17.18(77)	−1.08(19)	−1.00(51)<br>−7.05(70)	−4.27(100)<br>−10.64(97)	−3.22(59)<br>−9.91(60)
$V_{c_E}^{(1)}, V_{c_D}^{(1)}$<br>$V_{c_E}^{(l)}, V_{c_E}^{(t)}$	0.159	−0.98(55)	−8.13(84)<br>1.58(163)	−17.98(76)	−1.27(19)	−0.88(58)<br>−7.59(93)	−4.83(100)<br>−11.30(92)	−3.11(58)<br>−9.75(59)
$V_{c_E}^{(1)}, V_{c_E}^{(2)}$<br>$V_{c_D}^{(2)}, V_{c_E}^{(l)}$	0.165	−0.13(51)	−5.61(82)<br>0.18(89)	−17.34(78)	−0.38(19)	−0.06(52)<br>−4.26(84)	−4.11(99)<br>−10.87(89)	−2.78(60)<br>−8.29(61)
$V_{c_E}^{(1)}, V_{c_E}^{(2)}$<br>$V_{c_D}^{(1)}, V_{c_E}^{(l)}$	0.169	0.13(62)	−5.53(82)<br>−0.47(109)	−16.60(78)	−0.25(19)	−0.03(61)<br>−4.03(89)	−3.95(98)<br>−10.55(92)	−2.95(61)<br>−8.56(61)
$V_{c_E}^{(1)}, V_{c_D}^{(1)}$<br>$V_{c_D}^{(2)}, V_{c_E}^{(l)}$	0.185	0.28(74)	−6.09(82)<br>−1.80(153)	−16.35(78)	−0.14(19)	−0.06(76)<br>−3.58(118)	−3.84(99)<br>−10.57(93)	−2.91(60)<br>−8.38(61)
$V_{c_E}^{(1)}, V_{c_E}^{(2)}$<br>$V_{c_D}^{(1)}, V_{c_E}^{(t)}$	0.202	−1.29(53)	−11.38(82)<br>−2.41(227)	−17.90(77)	−0.77(19)	−0.53(49)<br>−5.39(56)	−0.12(98)<br>−7.17(93)	−2.50(60)<br>−8.73(61)
$V_{c_E}^{(2)}, V_{c_D}^{(1)}$<br>$V_{c_D}^{(2)}, V_{c_E}^{(l)}$	0.214	−0.19(51)	−5.64(82)<br>−0.88(168)	−17.82(77)	−0.52(19)	−0.28(49)<br>−4.69(66)	−3.76(99)<br>−10.79(86)	−2.47(60)<br>−7.90(61)
$V_{c_E}^{(1)}, V_{c_E}^{(2)}$<br>$V_{c_D}^{(2)}, V_{c_E}^{(t)}$	0.228	−1.39(62)	−10.34(82)<br>−0.83(236)	−18.04(78)	−0.97(19)	−0.63(61)<br>−6.09(72)	−1.30(98)<br>−8.51(89)	−2.44(62)<br>−8.64(62)
$V_{c_E}^{(1)}, V_{c_D}^{(2)}$<br>$V_{c_E}^{(l)}, V_{c_E}^{(t)}$	0.236	−1.12(58)	−8.74(87)<br>0.76(120)	−18.52(77)	−1.43(19)	−1.12(63)<br>−7.99(102)	−5.11(102)<br>−11.83(91)	−2.89(59)<br>−9.43(60)
$V_{c_E}^{(2)}, V_{c_D}^{(1)}$<br>$V_{c_E}^{(l)}, V_{c_E}^{(t)}$	0.244	−1.55(72)	−10.31(86)<br>−0.09(211)	−19.09(77)	−1.45(19)	−1.05(77)<br>−7.94(123)	−2.21(102)<br>−8.96(90)	−2.69(60)<br>−9.50(60)
$V_{c_D}^{(1)}, V_{c_D}^{(2)}$<br>$V_{c_E}^{(l)}, V_{c_E}^{(t)}$	0.245	−1.02(53)	−8.98(87)<br>−0.18(90)	−18.50(77)	−1.39(19)	−1.18(56)<br>−7.83(88)	−5.03(102)<br>−11.80(91)	−2.83(59)<br>−9.30(60)
$V_{c_E}^{(2)}, V_{c_D}^{(2)}$<br>$V_{c_E}^{(l)}, V_{c_E}^{(t)}$	0.258	−1.32(64)	−9.56(88)<br>0.31(141)	−18.82(78)	−1.49(19)	−1.19(69)<br>−8.15(112)	−4.37(103)<br>−11.17(92)	−2.79(61)<br>−9.41(61)
$V_{c_E}^{(1)}, V_{c_E}^{(2)}$<br>$V_{c_D}^{(1)}, V_{c_D}^{(2)}$	0.294	−0.69(53)	−6.18(82)<br>−0.76(90)	−18.28(76)	−0.41(19)	0.00(52)<br>−3.96(68)	−1.19(99)<br>−8.83(88)	−1.91(58)<br>−7.02(59)
$V_{c_E}^{(2)}, V_{c_D}^{(1)}$<br>$V_{c_D}^{(2)}, V_{c_E}^{(t)}$	0.298	−1.46(61)	−11.06(82)<br>−2.19(150)	−19.05(78)	−1.09(19)	−0.88(58)<br>−6.27(63)	−1.80(99)<br>−9.16(86)	−2.22(61)<br>−8.23(61)
$V_{c_E}^{(1)}, V_{c_D}^{(1)}$<br>$V_{c_D}^{(2)}, V_{c_E}^{(t)}$	0.367	−1.29(74)	−7.65(85)<br>−0.36(100)	−18.89(77)	−0.93(19)	−0.47(72)<br>−5.69(83)	−1.20(101)<br>−8.92(95)	−1.80(59)<br>−7.33(60)

Table S18. **Binding energy errors when including five additional 3N interactions.** Results for the RMSD over all calculated nuclear binding energies by using five additional 3N interactions as well as $V_{c_E}^{(0)}$ and $V_{c_D}^{(0)}$ to fit the nuclear binding energies for ^{3}H, ^{4}He, ^{7}Li, ^{8}Be, ^{9}Be, ^{10}Be, ^{10}B, ^{11}B, ^{12}C, ^{13}C, ^{14}C, ^{14}N, ^{15}N, ^{16}O, ^{17}O, and ^{18}O}. All energies are measured in MeV.

3N interactions in addition to $V_{c_E}^{(0)}, V_{c_D}^{(0)}$	RMSD B/A	$E_8^4 - 2E_4^2$	$E_{12,0_1^+}^6 - 3E_4^2$ / $E_{12,0_2^+}^6 - 3E_4^2$	$E_{16}^8 - 4E_4^2$	$E_6^2 - E_4^2$	$E_9^4 - E_8^4$ / $E_{10}^4 - E_8^4$	$E_{13}^6 - E_{12}^6$ / $E_{14}^6 - E_{12}^6$	$E_{17}^8 - E_{16}^8$ / $E_{18}^8 - E_{16}^8$
		0.10	−7.26	−14.42	−0.97	−1.67	−4.95	−4.14
			0.39			−8.47	−13.12	−12.19
$V_{c_E}^{(1)}, V_{c_E}^{(2)}, V_{c_D}^{(1)}$	0.102	−0.73(51)	−9.99(84)	−17.60(77)	−1.11(19)	−1.05(51)	−4.15(100)	−3.24(59)
$V_{c_E}^{(l)}, V_{c_E}^{(t)}$			−1.64(93)			−7.14(69)	−10.61(97)	−9.98(59)
$V_{c_E}^{(1)}, V_{c_E}^{(2)}, V_{c_D}^{(2)}$	0.144	−0.91(49)	−9.93(84)	−17.80(77)	−1.20(19)	−1.06(49)	−4.44(99)	−3.10(59)
$V_{c_E}^{(l)}, V_{c_E}^{(t)}$			−0.85(117)			−7.30(67)	−10.96(95)	−9.73(60)
$V_{c_E}^{(1)}, V_{c_E}^{(2)}, V_{c_D}^{(1)}$	0.185	0.02(57)	−5.59(82)	−17.22(77)	−0.40(19)	−0.23(55)	−3.80(99)	−2.66(60)
$V_{c_D}^{(2)}, V_{c_E}^{(l)}$			−1.24(179)			−4.46(72)	−10.67(86)	−8.16(61)
$V_{c_E}^{(2)}, V_{c_D}^{(1)}, V_{c_D}^{(2)}$	0.260	−1.30(61)	−9.63(87)	−18.85(77)	−1.48(19)	−1.20(65)	−4.39(102)	−2.78(60)
$V_{c_E}^{(l)}, V_{c_E}^{(t)}$			0.09(130)			−8.07(102)	−11.20(91)	−9.36(61)
$V_{c_E}^{(1)}, V_{c_D}^{(1)}, V_{c_D}^{(2)}$	0.270	−1.05(52)	−9.43(87)	−18.71(77)	−1.39(19)	−1.24(53)	−4.70(102)	−2.71(59)
$V_{c_E}^{(l)}, V_{c_E}^{(t)}$			−1.03(92)			−7.76(79)	−11.57(91)	−9.11(60)
$V_{c_E}^{(1)}, V_{c_E}^{(2)}, V_{c_D}^{(1)}$	0.337	−1.57(69)	−10.88(84)	−19.30(78)	−1.18(19)	−0.93(66)	−1.32(99)	−2.08(62)
$V_{c_D}^{(2)}, V_{c_E}^{(t)}$			−2.02(147)			−6.57(75)	−8.79(87)	−8.13(62)

Table S19. **Binding energy errors when including six additional 3N interactions.** Results for the RMSD over all calculated nuclear binding energies by using six additional 3N interactions as well as $V_{c_E}^{(0)}$ and $V_{c_D}^{(0)}$ to fit the nuclear binding energies for ^{3}H, ^{4}He, ^{7}Li, ^{8}Be, ^{9}Be, ^{10}Be, ^{10}B, ^{11}B, ^{12}C, ^{13}C, ^{14}C, ^{14}N, ^{15}N, ^{16}O, ^{17}O, ^{18}O, and ^{40}Ca. All energies are measured in MeV.

3N interactions in addition to $V_{c_E}^{(0)}, V_{c_D}^{(0)}$	RMSD B/A	$E_8^4 - 2E_4^2$	$E_{12,0_1^+}^6 - 3E_4^2$ / $E_{12,0_2^+}^6 - 3E_4^2$	$E_{16}^8 - 4E_4^2$	$E_6^2 - E_4^2$	$E_9^4 - E_8^4$ / $E_{10}^4 - E_8^4$	$E_{13}^6 - E_{12}^6$ / $E_{14}^6 - E_{12}^6$	$E_{17}^8 - E_{16}^8$ / $E_{18}^8 - E_{16}^8$
		0.10	−7.26	−14.42	−0.97	−1.67	−4.95	−4.14
			0.39			−8.47	−13.12	−12.19
$V_{c_E}^{(1)}, V_{c_E}^{(2)}, V_{c_D}^{(1)}$	0.079	−0.65(49)	−8.55(84)	−17.45(77)	−1.09(19)	−0.85(53)	−5.44(100)	−3.48(59)
$V_{c_D}^{(2)}, V_{c_E}^{(l)}, V_{c_E}^{(t)}$			0.99(168)			−7.19(83)	−11.79(106)	−10.23(60)
